# Mono‐Materials Created by Engineering a Continuum of P3HB Stereomicrostructures in a One‐Step Catalytic Process

**DOI:** 10.1002/anie.202518809

**Published:** 2025-11-26

**Authors:** Ethan C. Quinn, Celine R. Parker, Maëlle T. Gace, Minjung Lee, Shu Xu, Ethan Poppen, Zhen Zhang, Deepak K. Barange, Meltem Urgun‐Demirtas, Nicholas Rorrer, Eugene Y.‐X. Chen

**Affiliations:** ^1^ Department of Chemistry Colorado State University Fort Collins CO 80523–1872 USA; ^2^ Renewable Resources and Enabling Sciences Center National Renewable Energy Laboratory Golden CO 80401 USA; ^3^ Bio‐Optimized Technologies to keep Thermoplastics out of Landfills and the Environment (BOTTLE) Consortium Golden CO 80401 USA; ^4^ Department of Sustainable Materials and Processes Applied Materials Division Argonne National Laboratory Lemont IL 60439 USA; ^5^ Northwestern Argonne Institute of Science & Engineering Evanston IL 60208 USA

**Keywords:** All‐P3HB tapes, Biodegradable polymer, Mono‐materials, P3HB stereomicrostructure, Poly(3‐hydroxybutyrate)

## Abstract

Multi‐material products that combine multiple complementary polymers can create products with desired performance but present challenges to end‐of‐life (EoL) management. The emerging mono‐material product design based on a single polymer type addresses the fundamental EoL issue, but challenges of delivering vastly tunable material properties by the single polymer still remain. Here, we introduce a simple strategy to produce biodegradable poly(3‐hydroxybutyrate) (P3HB) materials with a wide range of material properties by engineering a stereomicrostructure continuum, achieved through polymerizing diastereomeric mixtures of *racemic* and *meso*‐dimethyl diolides at various feed ratios with a single catalyst. This one‐step, one‐pot process produces biodegradable P3HB mono‐materials ranging from rigid to flexible thermoplastics, to tough thermoplastic elastomers, to a pressure‐sensitive adhesive (PSA), which have been then combined to fabricate prototype all‐P3HB PSA tapes, demonstrating the feasibility of designing mono‐material products through engineering polymer stereomicrostructures.

## Graphical abstract

Diastereomeric mixtures of diolide were polymerized to P3HB mono‐materials that behave like thermoplastics, thermoplastic elastomers, and pressure‐sensitive adhesives, which were then combined to create all‐P3HB mono‐material tapes.

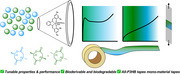

## Introduction

Multi‐material product design is a cornerstone of plastics innovation used to create products that synergistically combine properties of multiple materials.^[^ ^1^, ^2^, ^3^ ^]^ Sachet condiment packets are a great example as they are typically made up of five or more different polymers and additives all needed to achieve the necessary material properties. These polymers may include low‐density polyethylene used for sealing, high‐density polyethylene for strength, poly(ethylene‐*co*‐vinyl acetate) (EVA) as an adhesive tie layer, and poly(ethylene‐*co*‐vinyl alcohol) for oxygen and water barriers.^[^ ^1^ ^]^ Other multi‐material products include tapes, which typically are made from a backing of isotactic polypropylene (*it*‐PP), cellulose, or poly(vinyl chloride) and a pressure sensitive adhesive (PSA) portion typically made from rubber, acrylic, silicone, or urethane based polymers.^[^ ^4^, ^5^, ^6^, ^7^, ^8^, ^9^ ^]^ After use, these products cannot be efficiently mechanically recycled due to the immiscibility of these materials and the inherent difficulties of collection or separation. Even so, there have been advancements toward separating the components such as through solvent‐targeted recovery and precipitation^[^ ^10^, ^11^ ^]^ or compatibilization of these multi‐materials using universal dynamic crosslinkers^[^ ^12^, ^13^, ^14^, ^15^, ^16^ ^]^ and multi‐block copolymers.^[^ ^17^, ^18^, ^19^, ^20^ ^]^ These innovative approaches have great upsides but introduce chemical complexity and added cost to recycling processes and do not address the inherent problems most multi‐material products face: (fossil‐based) source and (environmentally recalcitrant) fate.^[^ ^21^, ^22^, ^23^, ^24^, ^25^, ^26^, ^27^ ^]^

To address the above challenges associated with multi‐material products, a transition toward single‐monomer‐based mono‐material product design is thought to be a potential solution.^[^ ^3^ ^]^ To achieve this goal, a polymer of a single chemical composition from a single monomer source needs to be tunable to exhibit the desired properties of various materials for different parts of a product or different products. One viable approach toward this goal involves engineering the stereomicrostructure of a polymer containing stereogenic centers.^[^ ^28^, ^29^, ^30^, ^31^ ^]^ An ideal candidate for this engineering is biodegradable poly(3‐hydroxybutyrate) (P3HB) comprising repeating units that bear a stereogenic tertiary carbon with a methyl group attached to it.

P3HB is the most common member of the large polyhydroxyalkanoate (PHA) family and is an extensively studied natural bio‐polyester.^[^ ^32^, ^33^, ^34^, ^35^, ^36^, ^37^, ^38^ ^]^ It is fully biodegradable in both managed and unmanaged conditions and, in its perfectly isotactic form, showcases an *it*‐PP‐like melting transition temperature (*T*_m_ = 175 °C – 180 °C) but is a brittle material (elongation at break, ε_B_ = 3% – 5%).^[^ ^32^ ^]^ When prepared biologically a stereo‐perfect isotactic form of P3HB is produced in the absolute (*R*) configuration (*sp*‐P3HB_B_). Synthetically this level of stereocontrol can also be achieved through the catalyzed ring‐opening polymerization (ROP) of the racemic eight‐membered dimethyl diolide (*rac*‐8DL^Me^) with bulky salen‐ligated yttrium catalysts which produced synthetic stereo‐perfect P3HB (*sp*‐P3HB_s_) with the probability of *meso* linkages (*P*_m_) >0.99 and percent *meso* triads ([*mm*]) >99%.^[^ ^39^ ^]^ Utilizing the four‐membered beta‐butyrolactone (BBL), advances in stereoselective ROP of *rac*‐BBL to highly syndiotactic (*st*)‐P3HB^[^ ^40^ ^]^ and *it‐*P3HB^[^ ^41^ ^]^ have also recently been accomplished. With the goal of creating biodegradable corollaries to plastic packaging materials, the diolide platform was then used to create iso‐rich (*ir*) or syndio‐rich (*sr*) P3HB from either *rac* or *meso*‐8DL^Me^ to create optically clear, mechanically ductile and tough thermoplastics with lower *T*_m_ values for more melt‐processable P3HB materials.^[^ ^42^, ^43^ ^]^ The *ir*‐P3HB can also be obtained from the ROP of *rac*‐BBL using salan‐ligated yttrium catalysts.^[^ ^44^ ^]^ Synergistic blending of *ir*‐ and *sr*‐P3HB with *sp*‐P3HB_s_ or *sp*‐P3HB_b_ was shown to significantly toughen the brittle *sp*‐P3HB material,^[^ ^42^ ^]^ which was especially the case with *ir*‐ and *sr*‐P3HB derived from 8DL^Me^. Furthering the scope of P3HB's capabilities, *sr*‐P3HB was found to be a strong adhesive, outperforming commercial glues like EVA.^[^ ^45^ ^]^ These overviewed examples show that stereomicrostructural engineering is a powerful tool to vastly tune P3HB's performance properties without the need to change its chemical composition, conforming to the principle of the mono‐material product design.

Polymerizations of racemic mixtures of BBL^[^ ^46^, ^47^ ^]^ and diastereomeric mixtures of 8DL^Me[^ ^48^ ^]^ can lead to stereodiblock P3HB, but this synthetic strategy affords limited tunability in the polymer material properties. It is desirable to develop a simple system that can be tuned to generate a continuum of stereo‐differentiated P3HBs bearing a wide range of material properties. We reasoned that the P3HBs produced at the extremes of the diastereomeric mixtures would produce tough thermoplastics, then at moderate diastereomeric mixtures more ductile, soft elastomers, and finally, in the middle of the spectrum, amorphous materials with tunable adhesive properties. Utilizing this broad range of material properties in a single product, such as PSA tapes, could serve as a representative of the mono‐material product design strategy. Notably, simplicity in chemical systems is of the utmost importance to embody the principles of green chemistry and to minimize waste and cost. Accordingly, this work designs a simple mono‐material polymer synthesis system by the catalyzed ROP of *rac*/*meso*‐8DL^Me^ mixtures, with the only change in the system being the diastereomeric ratio, which enabled generation of a large number stereodiverse P3HB mono‐materials possessing a continuum of stereomicrostructures to deliver vastly tunable material properties.

## Results and Discussion

### Creating a Continuum of P3HB Stereomicrostructures in One Step

**Figure 1:**
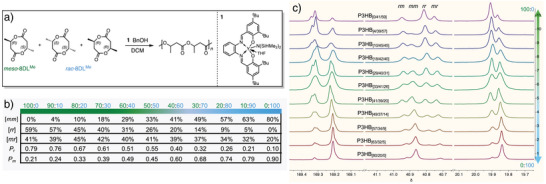
a) Overall reaction scheme for the synthesis of the new P3HBs described in this contribution. b) Table summarizing the stereomicrostructures of the P3HBs synthesized herein. Ratio of *meso*‐8DL^Me^ (green) to *rac*‐8DL^Me^ (blue) used to produce each P3HB is listed in the top row. c) Stacked ^13^C{^1^H} NMR spectra of the various P3HBs produced in this work to show a continuum of stereomicrostructures synthetically engineered.

Judicious catalyst selection is critical to produce a range of P3HB materials along a stereodiverse P3HB continuum, featuring stereo‐errors that can bring about desired mechanical performance. Utilizing a highly active yttrium catalyst generated from in situ alcoholysis of precatalyst **1** (Figure 1a) ligated with an achiral salen ligand with benzyl alcohol in a 1:1 ratio, *rac*‐8DL^Me^ was rapidly polymerized to P3HB with number‐average molar mass (*M*_n_) = 23.7 kg mol^−1^, dispersity (*Ð*) = 1.03, and [*mm*] = 80%.^[^ ^39^ ^]^ To assess the utility of this catalyst to produce P3HB with high molar mass (*M*_n_ >100 kg mol^−1^) from both *rac*‐ and *meso*‐8DL^Me^, the polymerization of *rac*‐ and *meso*‐8DL^Me^ was each tested, achieving full monomer conversion in <5 min to *ir*‐P3HB and *sr*‐P3HB, respectively (Tables S15 and S16). These controls formed a strong basis for the possibility of using **1** to produce a continuum of P3HB stereomicrostructures from diastereomeric mixtures of *rac*‐8DL^Me^ and *meso*‐8DL^Me^ in different ratios, due to its high activity toward both monomers resulting in P3HBs with catalyst‐monomer specific stereomicrostructures. Accordingly, a spectrum of P3HB stereomicrostructures were indeed produced by modulating the diastereomeric feed ratio of *rac* and *meso*‐8DL^Me^ in integers of 10 (Figure 1b). All the diastereomeric mixtures were quantitatively polymerized to yield 11 different P3HB stereomicrostructures with high *M*_n_ (>100 kg mol^−1^) and low *Ð* (<1.3) values (Table 1). As slight changes in triad sequence distribution can lead to great impacts on viscoelastic properties,^[^ ^42^, ^45^ ^]^ it is important to perform detailed analysis of P3HB stereomicrostructures (all three triads) by ^13^C{^1^H} NMR. As shown in Figure 1c, the percent triads can be tracked through integration of the methylene region from *δ* 40.78 to 40.98 ppm. Previous work characterizing the mechanism of this polymerization system has showed the absence of chain shuttling and epimerization in these systems.^[^ ^39^, ^48^ ^]^ As a result, P3HB_[0/41/59]_ is rich in syndiotactic [*rr*] content (59%) and completely devoid of any isotactic [*mm*] triad. The reverse is true for P3HB_[80/20/0]_ which is rich in [*mm*] content (80%) and devoid of any [*rr*] triad. Consequently, this synthetic strategy allowed installation of opposite triads to P3HB that are inaccessible when the diastereomers are segregated, providing an access to “in‐between” stereomicrostructures.

**Table 1:** Physical properties of P3HBs (subscript values denote [*mm*/*mr*/*rr*] percentages) prepared in this study.^a)^

Entry	*meso*:*rac* (8DL^Me^)	P3HB	*M*_n_ (kg mol^−1^)^a^ ^)^	*Ð* ^a^ ^)^ (*M*_w_/*M*_n_)	*T*_m_ (°C)	Δ*H*_f_ (J g^−1^)	*T*_g_ (°C)	*X*_c_ (%)	*σ*_B_ (MPa)	*ε*_B_ (%)	*E* (MPa)	*U*_T_ (MJ m^−3^)
1^b)^	100:0	P3HB_[0/41/59]_	216	1.29	121	38.7	13	24	31.4	354	306	77.0
2^b)^	90:10	P3HB_[4/39/57]_	142	1.16	109	31.3	7.2	21	31.0	476	98.6	93.8
3^b)^	80:20	P3HB_[10/45/45]_	126	1.01	105	16.9	7.3	12	16.4	530	14.6	49.6
4	70:30	P3HB_[18/42/40]_	111	1.01	71.2	28.3	7.2	19	15.1	675	15.2	55.6
5^c)^	60:40	P3HB_[29/40/31]_	105	1.16	–	–	0.1	–	–	–	–	–
6	50:50	P3HB_[33/41/26]_	144	1.10	–	–	5.3	–	–	–	–	–
7	40:60	P3HB_[41/39/20]_	105	1.06	–	–	4.9	–	–	–	–	–
8	30:70	P3HB_[49/37/14]_	155	1.18	73.9	2.6	2.7	1.8	4.1	414	1.37	8.3
9	20:80	P3HB_[57/34/9]_	195	1.21	71.0	16.6	0.9	11	10.7	774	26.3	33.4
10	10:90	P3HB_[63/32/5]_	102	1.03	93.7	41.0	5.6	28	9.5	552	294	35.5
11^d)^	0:100	P3HB_[80/20/0]_	106	1.04	108	26.8	4.0	18	22.2	375	800	60.4

^a)^ Absolute number‐average molar mass (*M*_n_), and dispersity (*Ð* = *M*_w_/*M*_n_) determined by size‐exclusion chromatography (SEC) coupled with a Wyatt DAWN HELEOS II multi (18)‐angle light scattering detector and a Wyatt Optilab TrEX dRI detector and performed at 40 °C in chloroform.

^b)^ Thermal data collected on second heating scan, whereas all others were collected on first heating scan.

^c)^ Data included was previously reported.^[^ ^45^ ^]^

^d)^ Data included was previously reported.^[^ ^42^ ^]^

As the feed ratio was gradually shifted from pure *meso‐*8DL^Me^ to pure *rac‐*8DL^Me^, the amounts of each triad changed incrementally, shifting in dominance from [*rr*] to [*mm*] content, respectively (Figure 1b). For the first three P3HBs, P3HB_[0/41/59]_, P3HB_[4/39/57]_, and P3HB_[10/45/45]_, [*rr*] makes up the greatest percentage of their triads, but as the *rac*‐8DL^Me^ content increased by 10% in each feed ratio, the [*rr*] content incrementally decreased. When the *rac*:*meso*‐8DL^Me^ ratio reached 70:30, stereo‐randomness came to dominate when the [*mr*] content, at 42%, rose higher than the [*rr*] content in P3HB_[18/42/40]_. In the subsequent P3HB_[29/40/31]_ and P3HB_[33/41/26]_, the [*mr*] content continued to dominate imbuing a high amount of randomness in their stereomicrostructures. As the feed ratio reached 60:40, the [*mm*] content of P3HB_[41/39/20]_ was increased to be greater than the [*mr*] content at 39%. For the remaining samples, P3HB_[49/37/14]_, P3HB_[57/34/9]_, P3HB_[63/32/5]_, and P3HB_[80/20/0]_, the [*mm*] content maintained the largest percentage sequence contribution toward the stereomicrostructure, and the [*rr*] content decreased incrementally to zero. Overall, the above results showed dialing in the monomer's diastereomeric ratio effectively created a continuum of stereomicrostructures of P3HB.

### Stereomicrostructure‐Differentiated Thermal and Mechanical Properties

The thermal properties varied from amorphous materials (P3HB_[29/40/31]_, P3HB_[33/41/26]_, and P3HB_[41/39/20]_) with glass transition temperatures (*T*_g_’s) ranging from 0.1 °C and 5.3 °C, to low melting, low crystallinity P3HBs (P3HB_[10/45/45]_, P3HB_[18/42/40]_, P3HB_[49/37/14]_, and P3HB_[57/34/9]_) exhibiting *T*_m_’s ranging from 71.0 °C to 105 °C, to higher melting, higher crystallinity P3HBs from the extremes of the continuum (P3HB_[0/41/59]_, P3HB_[4/39/57]_, P3HB_[63/32/5]_, and P3HB_[80/20/0]_) exhibiting *T*_m_’s ranging from 93.7 °C to 121 °C (Table 1; Figures S24–S33). The P3HB with the highest *T*_m_ of this series is P3HB_[0/41/59]_ at *T*_m_ = 121 °C and Δ*H*_f_ (heat of fusion) = 38.7 J g^−1^, which is a semi‐crystalline polymer with a percent crystallinity (*X*_c_) of 24%, a crystallization temperature (*T*_c_) of 74.0 °C, and heat of crystallization (Δ*H*_c_) = 34.4 J g^−1^. The *T*_m_ decreased with the decreasing [*rr*] content in P3HB_[4/39/57]_, displaying a *T*_m_ of 109 °C (Δ*H*_f_ = 31.3 J g^−1^), an *X*_c_ of 21%, and a *T*_g_ of 7.2 °C. The Δ*H*_f_ was halved in P3HB_[10/45/45]_ to 16.9 J g^−1^ (*X*_c_ = 12%), whereas its *T*_m_ (105 °C) and *T*_g_ (7.3 °C) are similar to the previous sample. As the [*rr*] content continued to decrease, the *T*_c_ became undetectable in the remaining samples and *T*_m_ values were only recordable from the first heating scans. The first example of this trend was *sr*‐P3HB_[18/42/40]_ which exhibited a *T*_m_ of 71.2 °C (Δ*H*_f_ = 28.3 J g^−1^), an *X*_c_ of 19%, and a *T*_g_ of 7.2 °C. Upon reaching P3HB_[29/40/31]_, it became an amorphous material for which only a *T*_g_ was observed. This behavior held true for P3HB_[33/41/26]_ and P3HB_[41/39/20]_ which exhibited *T*_g_ values of 5.3 °C and 4.9 °C, respectively. In the remaining samples, the semi‐crystalline character returns, although *T*_c_ values remained undetected. This trend was first shown in P3HB_[49/37/14]_ which exhibited a *T*_m_ of 73.9 °C (Δ*H*_f_ = 2.6 J g^−1^), an *X*_c_ of 1.8%, and a *T*_g_ of a 2.7 °C. The semi‐crystalline character continued to increase with increasing the [*mm*] content, with P3HB_[57/34/9]_ displaying a *T*_m_ of 71.0 °C (Δ*H*_f_ = 16.6 J g^−1^), an *X*_c_ of 11%, and a *T*_g_ of 0.9 °C. Last, P3HB_[63/32/5]_ exhibited a *T*_m_ of 93.7 °C (Δ*H*_f_ = 41.0 J g^−1^), an *X*_c_ of 28%, and a *T*_g_ of 5.6 °C.

**Figure 2:**
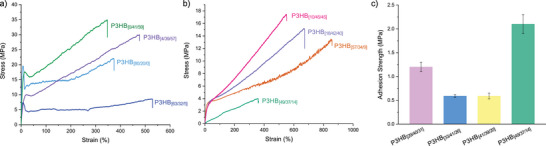
a) Stress–strain overlays of thermoplastic P3HBs. b) Stress–strain overlays of thermoplastic elastomeric P3HBs. c) Adhesion strengths of adhesive P3HBs on aluminum substrates (error bars correspond to the standard deviation of 4 replicates).

Next, semi‐crystalline P3HBs were subjected to uniaxial tensile testing using dog‐bone shaped samples (Figures S35–S41 and Tables S4–S10). The materials on the extreme ends of the stereomicrostructure spectrum (P3HB_[0/41/59],_ P3HB_[4/39/57]_, P3HB_[80/20/0]_, and P3HB_[63/32/5]_) showed tensile properties characteristic of thermoplastics (Figure 2a), whereas materials in lesser extremes (P3HB_[10/45/45],_ P3HB_[18/42/40]_, P3HB_[49/37/14]_, and P3HB_[57/34/9]_) behaved like thermoplastic elastomers (Figure 2b). All materials tested had *M*_n_ > 10x entanglement molecular weight (*M*_e_). The *M*_e_ was found to be between 6,210 and 7,417 g mol^−1^ for the three adhesive polymers tested (Table S2), indicating significant chain entanglement which is necessary for adequate mechanical properties.

Strong and tough thermoplastic P3HB_[0/41/59]_ exhibited an ultimate tensile strength (*σ*_B_) of 31.4 ± 3.1 MPa, *ε*_B_ of 354% ± 6.5%, Young's modulus (*E*) of 306 ± 34 MPa, and high toughness (*U*_T_) of 77.0 ± 6.4 MJ m^−3^. When the crystallinity was reduced in P3HB_[4/39/57]_, the material showed an enhanced *ε*_B_ of 476% ± 5.3% and thus an increased *U*_T_ of 93.8 ± 3.7 MJ m^−3^. With a further reduction in the [*rr*] content, P3HB_[10/45/45]_ became softened to be more elastomeric, exhibiting no yield point but an enhanced *ε*_B_ to 530% ± 72%, a reduced *σ*_B_ to 16.4 ± 2.1 MPa and *E* to 14.6 ± 5.6 MPa. Among the lesser [*rr*] content materials, P3HB_[18/42/40]_ exhibited the highest *ε*_B_ of 675% ± 1.5%. As there is not an observable yield point, P3HB_[18/42/40]_ was subjected to hysteresis and exhibited an energy loss coefficient of 3.5 MJ m^−3^ at 25% strain and 17.2 MJ m^−3^ at 50% strain with good elastic recovery after 10 cycles (Figures S42 and S43). With no [*rr*] content, P3HB_[80/20/0]_ is a tough thermoplastic,^[^ ^42^ ^]^ and P3HB_[63/32/5]_ also reflected this characteristic with *ε*_B_ = 552% ± 43%, *E* = 294 ± 8.4 MPa, and *U*_T_ = 35.5 ± 7.6 MJ m^−3^. With the inclusion of more stereo‐errors in P3HB_[57/34/9]_, it behaved more like a thermoplastic elastomer, exhibiting no yield point and elongating to a large strain without fracture (*ε*_B_ = 774% ± 192%). The tensile performance of P3HB_[49/37/14]_ was not as enhanced by inclusion of the more atactic [*mr*] content as observed in the P3HBs with the higher [*rr*] content, and it exhibited a reduced *ε*_B_ of 414% ± 96%. Overall, the continuum of the P3HB stereomicrostructures corresponded to the spectrum of mechanical properties, expanding the design space for all‐P3HB mono‐materials across a range of rigid to flexible thermoplastics, tough plastomers, and weak elastomers.

We reported previously that P3HB_[29/40/31]_ exhibited a modest adhesion strength of 1.2 ± 0.1 MPa toward aluminum substrates.^[^ ^45^ ^]^ In comparison, P3HB_[33/41/26]_ and P3HB_[41/39/20]_ with a similar [*mr*] content but a higher [*mm*]/[*rr*] ratio of >1 displayed a lower adhesion strength of 0.59 MPa (Figure 2c and Table S12). On the other hand, when the [*mm*]/[*rr*] ratio reached to ∼2 while keeping the [*mr*] content approximately the same, namely P3HB_[49/37/14]_, its adhesion strength was enhanced to 2.1 ± 0.2 MPa (Figure 2c and Table S12). Hence, the above results further highlighted the high sensitivity of P3HB's adhesion strength to its stereomicrostructure.^[^ ^44^ ^]^

### Rheological Properties of PSA P3HB

**Figure 3:**
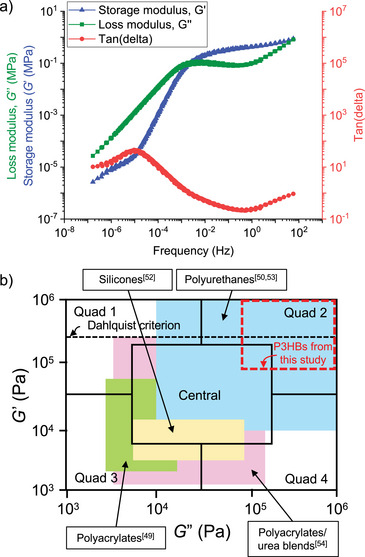
a) Rheological time‐temperature superposition (TTS) curve of P3HB_[33/41/26]_ (reference temperature = 303 K). b) Chang's viscoelastic window (VW) of different types of pressure sensitive adhesives (PSAs),^[^ ^49^, ^50^, ^52^, ^53^, ^54^ ^]^ compared to P3HB_[29/40/31]_, P3HB_[33/41/26]_, and P3HB_[41/39/20]_ of this work.

Viscoelastic behaviors of P3HB_[29/40/31]_, P3HB_[33/41/26]_, and P3HB_[41/39/20]_ were examined via shear rheology to obtain master curves of dynamic storage modulus *G*′ and loss modulus *G*″ (Figure 3a and Table S1). Figure 3a presents the master curve of P3HB_[33/41/26]_, which showed favorable adhesive properties by the presence of both a creep region (*G*″>*G*′, below 0.01Hz) and tack regions (*G*′>*G*″, in between 0.01 and 100Hz). These characteristics are also observed in P3HB_[29/40/31]_ and P3HB_[41/39/20]_ (Figure S1 and Table S1). Figure 3b shows that P3HB_[29/40/31]_, P3HB_[33/41/26]_, and P3HB_[41/39/20]_ all appear in quadrant 2 of Chang's Viscoelastic Window (VW)^[^ ^49^ ^]^ where they have great characteristics (i.e., high *G*′ and high *G*″) for high shear PSAs in high‐performance tapes. Among other types of PSAs, the P3HB sample range in Chang's VW is comparable to thermoplastic polyurethanes.^[^ ^50^ ^]^ Further viscosity measurements and rheological testing were also carried out (Figures S2 and S3; Tables S2 and S3). The temperature sweep measurements (Figure S2) showed that both *G*′ and *G*″ increased as the [*rr*] content increased. The crossover point between 50 °C – 60 °C indicated the highest application temperature as adhesives. Shear thinning behavior was observed in all three samples tested through viscosity measurements (Figure S3). The viscosity drop was abrupt due to the highly monodisperse nature of the samples, indicating highly aligned polymer chains along the direction of flow.^[^ ^51^ ^]^ The drop occurs due to disentanglement of the polymer chain upon increased shear rate, and the highly aligned polymer chains are more prone to be disentangled compared to less aligned polymer chains.

### Fabrication of All‐P3HB Mono‐Material Tapes

**Figure 4:**
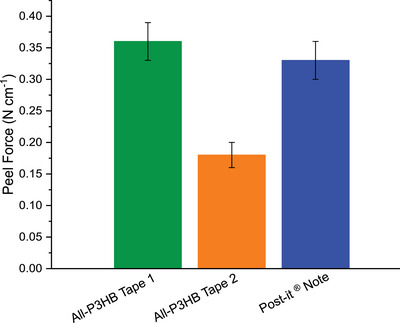
Peel strength of all‐P3HB tapes in comparison with a Post‐It^®^ note.

The successful synthesis of P3HBs with a continuum of stereomicrostructures from the mixture of *rac* and *meso*‐8DL^Me^ in different ratios and rheological validation of the viscoelastic properties for adhesives laid a foundation for creating an all‐P3HB tape as a prototype mono‐material product. Accordingly, two all‐P3HB tapes were fabricated by first preparing a thin backing layer in the hot press and then applying a thin layer of adhesive P3HB in the hot press. Tape 1 was created using semicrystalline P3HB_[10/45/45]_ (*T*_m_ = 105 °C, Δ*H*_f_ = 16.9 J g^−1^) as the backing and amorphous P3HB_[41/39/20]_ as the PSA making an elastomeric tape with a moderate peel strength of 0.35 ± 0.03 N mm^−1^ (Figure 4; a photo of the tape is provided in Figure S44). Tape 2 was created using more crystalline P3HB_[0/41/59]_ (*T*_m_ = 121 °C, Δ*H*_f_ = 38.7 J g^−1^) as the backing and the same amorphous P3HB_[41/39/20]_ as the PSA making an elastomeric tape, but it exhibited a lower peel strength of 0.18 ± 0.02 N mm^−1^. Notably, all‐P3HB tape 1 showed the similar peel strength to a Post‐It^®^ note based on an in‐house peel test (Figure 4 and Table S13). Further engineering of the peel strength of the all‐P3HB tapes is possible, but this preliminary demonstration provided a proof‐of‐concept for an all‐P3HB mono‐material. To demonstrate the recyclability of the mono‐material tapes created, Tape 1 was subjected to solvent recycling^[^ ^42^ ^]^ and the resulting semi‐crystalline blend showed improved material properties (*σ*_B_ = 24.7 ± 2.3 MPa, *ε*_B_ = 574% ± 51%) compared to the tape backing used, indicating that these products can be recycled and further used (Figures S34 and S45, Table S11).

### Stereomicrostructure‐Dependent Biodegradation

**Figure 5:**
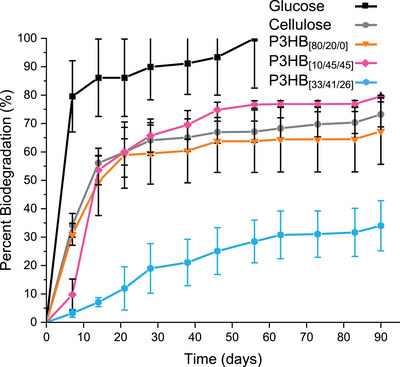
Freshwater biodegradation (ISO 14851 standard) of P3HB_[80/20/0]_, P3HB_[10/45/45]_, and P3HB_[33/41/26]_ after 90 days.

The biodegradability of new P3HB stereomicrostructures was tested to evaluate their biodegradation end‐of‐life (EoL) option. Previous work has shown the freshwater biodegradation of *sr*‐P3HB^[^ ^43^ ^]^ following the ISO 14851 standard exhibited ∼54% degradation in 90 days and was projected using first‐order kinetics to reach ∼90% degradation in ∼383 days. Using the same testing procedure, P3HB_[10/45/45]_ and P3HB_[80/20/0]_ showed faster freshwater biodegradation of ∼80% and ∼67% in 90 days, respectively, whereas P3HB_[33/41/26]_ showed slower biodegradation of ∼34% in the same time frame, indicating that the overall stereomicrostructures (i.e., all three triad sequences) play a role on the P3HB biodegradation rate and semi‐crystalline P3HB degrades faster than amorphous P3HB (Figure 5). More specifically, for the two semicrystalline P3HB samples, the P3HB with the higher percentage of the heterotactic triad [*mr*] sequence biodegrades faster, P3HB_[10/45/45]_ > P3HB_[80/20/0]_. On the other hand, the amorphous sample where the [*mr*] sequence dominates homochiral sequences, P3HB_[33/41/26]_, appeared rubbery and degraded the slowest. Using a first order kinetic model, P3HB_[10/45/45]_ and P3HB_[80/20/0]_ were predicted to reach 90% biodegradation in 131 and 273 days, respectively, whereas P3HB_[33/41/26]_ was predicted to reach the 90% degradation in 529 days (Table S14). Overall, the results that the P3HB containing the highest percentages of unnatural triad sequences ([*mr*] and [*rr*]), P3HB_[10/45/45]_, degrades first showed that P3HB materials with synthetically created new stereomicrostructures designed for tailored materials performance did not compromise the biodegradability of P3HB, ensuring the biodegradation EoL option for synthetic P3HBs with unnatural stereomicrostructures.

## Conclusions

This work establishes a simple mono‐material polymer synthesis system that utilizes the catalyzed ROP of varied ratios of *rac*/*meso*‐8DL^Me^ mixtures, creating a large number stereodiverse P3HB materials possessing a continuum of stereomicrostructures that possess vastly tunable material properties. The power of expedient access to P3HB mono‐materials via stereomicrostructural engineering was exemplified in this work as materials that ranged three orders of magnitude in material properties can be synthesized in a simple fashion. Rigid to flexible thermoplastics, tough thermoplastic elastomers, and PSAs were produced and subsequently utilized to fabricate all‐P3HB mono‐material tapes. Also notably, this work addresses a fundamental knowledge gap in the PHA literature as P3HB with “in‐between” stereomicrostructures were accessed for the first time, and it also furthers our understanding of the fundamental relationship between P3HB's stereomicrostructure and its material properties.

## Supporting Information

The authors have cited additional references within the Supporting Information.^[^ ^55^, ^56^, ^57^, ^58^, ^59^, ^60^ ^]^

## Conflict of Interests

The authors declare no conflict of interest.

## Supporting information



Supporting Information

## Data Availability

The data that support the findings of this study are available in the Supporting Information of this article.
