# Supplementary material for: Mono‐Materials Created by Engineering a Continuum of P3HB Stereomicrostructures in a One‐Step Catalytic Process
Source: Angew Chem Int Ed Engl. 2025 Nov 26;65(3):e18809. doi: 10.1002/anie.202518809 (PMC12811665; doi:10.1002/anie.202518809)
Supplement: Supplementary file 1 — Supporting Information [file ANIE-65-e18809-s001.pdf]

Electronic Supplementary Information

Mono-Materials Created by Engineering a Continuum of P3HB Stereomicrostructures in a One-Step Catalytic Process

Ethan C. Quinn,†[a] Celine R. Parker,†[a] Maëlle T. Gace,[a] Minjung Lee,[b,c] Shu Xu,[d,e] Ethan Poppen,[a] Zhen Zhang,[a] Deepak K. Barange,[a] Meltem Urgun-Demirtas,[d] Nicholas Rorrer,[b,c] and Eugene Y.-X. Chen*[a]

---

[a] Dr. E. C. Quinn, Dr. C. R. Parker, M. T. Gace, E. Poppen, Prof. Dr. Z. Zhang, Dr. D. K. Barange, Prof. Dr. E. Y.-X. Chen
Department of Chemistry, Colorado State University
Fort Collins, CO 80523–1872 (USA)
E-mail: eugene.chen@colostate.edu

[b] Dr. M. Lee, Dr. N. Rorrer
Renewable Resources and Enabling Sciences Center, National Renewable Energy Laboratory
Golden, CO 80401 (USA)

[c] Dr. M. Lee, Dr. N. Rorrer
Bio-Optimized Technologies to keep Thermoplastics out of Landfills and the Environment (BOTTLE) Consortium
Golden, CO 80401 (USA)

[d] Dr. S. Xu, Dr. Prof. M. Urgun-Demirtas
Department of Sustainable Materials and Processes, Applied Materials Division, Argonne National Laboratory
Lemont IL 60439 (USA)

[e] Dr. S. Xu
Northwestern Argonne Institute of Science & Engineering
Evanston IL 60208 (USA)

†These authors contributed equally to this work

Table of Contents

Materials and Methods 5
Materials 5
General polymerization procedure 5
NMR spectroscopy 5
Size-exclusion chromatography 5
Differential scanning calorimetry 5
Mechanical testing 5
Recycling of Mono-Material Products 6
Dynamic Mechanical Analysis (DMA) 6
Rheological Analysis 6
Biodegradation Testing 7
End of lifetime estimation of polymer samples 8
Supplementary Figures 9
Figure S1. Time-temperature superposition master curves of P3HB polymers. 9
Figure S2. Temperature sweep measurements of P3HB polymers. 9
Figure S3. Steady-state shear viscosity of P3HB polymers. 9
Figure S4. SEC trace of $P3HB_{[0/41/59]}$ (M_n = 216 kg mol^{-1}, M_w = 277 kg mol^{-1}, $Đ$ = 1.29). 10
Figure S5. SEC trace of $P3HB_{[4/39/57]}$ (M_n = 142 kg mol^{-1}, M_w = 165 kg mol^{-1}, $Đ$ = 1.16). 10
Figure S6. SEC trace of $P3HB_{[10/45/45]}$ (M_n = 126 kg mol^{-1}, M_w = 126 kg mol^{-1}, $Đ$ = 1.01). 11
Figure S7. SEC trace of $P3HB_{[18/42/40]}$ (M_n = 111 kg mol^{-1}, M_w = 113 kg mol^{-1}, $Đ$ = 1.01). 11
Figure S8. SEC trace of $P3HB_{[29/40/31]}$ (M_n = 105 kg mol^{-1}, M_w = 122 kg mol^{-1}, $Đ$ = 1.16). 12
Figure S9. SEC trace of $P3HB_{[33/41/26]}$ (M_n = 144 kg mol^{-1}, M_w = 157 kg mol^{-1}, $Đ$ = 1.10). 12
Figure S10. SEC trace of $P3HB_{[41/39/20]}$ (M_n = 105 kg mol^{-1}, M_w = 111 kg mol^{-1}, $Đ$ = 1.06). 13
Figure S11. SEC trace of $P3HB_{[49/37/14]}$ (M_n = 155 kg mol^{-1}, M_w = 183 kg mol^{-1}, $Đ$ = 1.18). 13
Figure S12. SEC trace of $P3HB_{[57/34/9]}$ (M_n = 195 kg mol^{-1}, M_w = 236 kg mol^{-1}, $Đ$ = 1.21). 14
Figure S13. SEC trace of $P3HB_{[63/32/5]}$ (M_n = 102 kg mol^{-1}, M_w = 105 kg mol^{-1}, $Đ$ = 1.03). 14
Figure S14. ^{13}C NMR Spectrum ($CDCl_3$, 23 °C) of $P3HB_{[0/41/59]}$ with inset of methylene region. 15
Figure S15. ^{13}C NMR Spectrum ($CDCl_3$, 23 °C) of $P3HB_{[4/39/57]}$ with inset of methylene region. 15
Figure S16. ^{13}C NMR Spectrum ($CDCl_3$, 23 °C) of $P3HB_{[10/45/45]}$ with inset of methylene region. 16
Figure S17. ^{13}C NMR Spectrum ($CDCl_3$, 23 °C) of $P3HB_{[18/42/40]}$ with inset of methylene region. 16
Figure S18. ^{13}C NMR Spectrum ($CDCl_3$, 23 °C) of $P3HB_{[29/40/31]}$ with inset of methylene region. 17
Figure S19. ^{13}C NMR Spectrum ($CDCl_3$, 23 °C) of $P3HB_{[33/41/26]}$ with inset of methylene region. 17
Figure S20. ^{13}C NMR Spectrum ($CDCl_3$, 23 °C) of $P3HB_{[41/39/20]}$ with inset of methylene region. 18
Figure S21. ^{13}C NMR Spectrum ($CDCl_3$, 23 °C) of $P3HB_{[49/37/14]}$ with inset of methylene region. 18
Figure S22. ^{13}C NMR Spectrum ($CDCl_3$, 23 °C) of $P3HB_{[57/34/9]}$ with inset of methylene region. 19
Figure S23. ^{13}C NMR Spectrum ($CDCl_3$, 23 °C) of $P3HB_{[63/32/5]}$ with inset of methylene region. 19
Figure S24. DSC curve of $P3HB_{[0/41/59]}$ (M_n = 216 kg mol^{-1}, $Đ$ = 1.29). 2nd heating scan and cooling scan. 20
Figure S25. DSC curve of $P3HB_{[4/39/57]}$ (M_n = 142 kg mol^{-1}, $Đ$ = 1.16). 2nd heating scan and cooling scan. 20

Figure S26. DSC curve of P3HB$_{[10/45/45]}$ (M_n = 126 kg mol^{-1}, $Đ$ = 1.01). 2nd Heating scan and cooling scan.........21
Figure S27. DSC curve of P3HB$_{[18/42/40]}$ (M_n = 111 kg mol^{-1}, $Đ$ = 1.01). 1st heating scan and cooling scan...........21
Figure S28. DSC curve of P3HB$_{[29/40/31]}$ (M_n = 105 kg mol^{-1}, $Đ$ = 1.16). 1st heating scan and cooling scan...........22
Figure S29. DSC curve of P3HB$_{[33/41/26]}$ (M_n = 144 kg mol-1, $Đ$ = 1.10). 1st heating scan and cooling scan. 22
Figure S30. DSC curve of P3HB$_{[41/39/20]}$ (M_n = 105 kg mol^{-1}, $Đ$ = 1.06). 1st heating scan and cooling scan. 23
Figure S31. DSC curve of P3HB$_{[49/37/14]}$ (M_n = 155 kg mol^{-1}, $Đ$ = 1.18). 1st heating scan and cooling scan. DSC artifact observed from 92-100 °C. 23
Figure S32. DSC curve of P3HB$_{[57/34/9]}$ (M_n = 195 kg mol^{-1}, $Đ$ = 1.21). 1st heating scan and cooling scan. DSC artifact observed from 92-97 °C. 24
Figure S33. DSC curve of P3HB$_{[63/32/5]}$ (M_n = 102 kg mol^{-1}, $Đ$ = 1.03). 1st heating scan and cooling scan............24
Figure S34. DSC curve of recycled Tape 1. Black line is the 1st heating scan and cooling scan; blue line is the second heating scan. 25
Figure S35.Triplicate stress-strain curves of P3HB$_{[0/41/59]}$ (M_n = 216 kg mol^{-1}, $Đ$ = 1.29). 26
Figure S36. Triplicate stress-strain curves of P3HB$_{[4/39/57]}$ (M_n = 142 kg mol^{-1}, $Đ$ = 1.16). 27
Figure S38. Triplicate stress-strain curves of P3HB$_{[18/42/40]}$ (M_n = 111 kg mol^{-1}, $Đ$ = 1.01). 29
Figure S39. Triplicate stress-strain curves of P3HB$_{[49/37/14]}$ (M_n = 155 kg mol^{-1}, $Đ$ = 1.18). 30
Figure S40. Triplicate stress-strain curves of P3HB$_{[57/34/9]}$ (M_n = 195 kg mol^{-1}, $Đ$ = 1.21). 31
Figure S41. Triplicate stress-strain curves of P3HB$_{[63/32/5]}$ (M_n = 102 kg mol^{-1}, $Đ$ = 1.03). 32
Figure S42. Continuous loading-unloading tensile curves of P3HB$_{[18/42/40]}$ at 25% strain (black) and 50% strain (green); η = energy loss coefficient. 33
Figure S43. Hysteresis of P3HB$_{[18/42/40]}$ with 10 loading-unloading cycles at a strain of 25% without any resting time; run in triplicate on three distinct samples. 34
Figure S44. Photo of Tape 1 mounted on an Instron for peel testing. 35
Figure S45. Triplicate stress-strain curves of recycled Tape 1. 36
Supplementary Tables 37
Table S1. Shift factors analyzed from the master curve. 37
Table S2. Plateau modulus and the entanglement molecular weight analyzed from master curve. 37
Table S3. Carreau model parameters fitted by the viscosity measurement. 37
Table S4. Triplicate tensile data for P3HB$_{[0/41/59]}$. 37
Table S5. Triplicate tensile data for P3HB$_{[4/39/57]}$. 37
Table S6. Triplicate tensile data for P3HB$_{[10/45/45]}$. 37
Table S7. Triplicate tensile data for P3HB$_{[18/42/40]}$. 38
Table S8. Triplicate tensile data for P3HB$_{[49/37/14]}$. 38
Table S9. Triplicate tensile data for P3HB$_{[57/34/9]}$. 38
Table S10. Triplicate tensile data for P3HB$_{[63/32/5]}$. 38
Table S11. Triplicate tensile data for recycled Tape 1. 38
Table S12. Adhesion data of adhesive P3HBs. 39
Table S13. Peel testing data of manufactured tapes. 39
Table S14. Summary of estimated lifetime of P3HB samples in freshwater environments using first order kinetic model. 39
Table S15. Result of *meso*-8DL^{Me} polymerization[a]. 39

Table S16. Result of *rac*-8DL^{Me} polymerization[a]. 39
Table S17. Result of *meso*-8DL^{Me}/*rac*-8DL^{Me} copolymerizations[a]. 40
Supplementary Note 40
Bibliography 40

Materials and Methods

Materials

All monomers and catalysts employed in this study were synthesized according to literature procedures.[39, 48] Benzyl alcohol (BnOH) was obtained from TCI Chemicals and HPLC-grade dichloromethane was obtained from Acros Organics and each of them were stirred for 12 h on activated calcium hydride and distilled before use. All syntheses and manipulations of air- and moisture-sensitive materials were carried out in flamed Schlenk-type glassware on a dual-manifold Schlenk line, on a high-vacuum line, or in an N_2-filled glovebox. Aluminum substrates used for adhesion tests were purchased from Home Depot and used as received.

General polymerization procedure

Polymerizations were performed in glass reactors inside an inert N_2 glovebox at ambient temperature (~23 °C). The reactor was first charged with a predetermined amount of monomer and dichloromethane (1.5 M, DCM), and a mixture of catalyst and initiator in solvent was stirred at ambient temperature for 10 minutes in another reactor. The polymerization was initiated by rapid addition of the 0.15 mol% catalyst solution to the monomer solution. After a desired time, a 0.01 mL of aliquot was taken from the reaction mixture and quenched with a 5% solution of benzoic acid in $CDCl_3$ for ^{1}H NMR analysis to obtain the percent monomer conversion data. The polymerization was later quenched by addition of HCl/methanol (5% solution, 20:1 ratio of polymerization solution to quenching solvent). The quenched mixture was then filtered, redissolved in a minimal amount of dichloromethane, and precipitated into an excess amount of cold methanol while stirring. This process was repeated a total of three times and the polymer was then dried in a vacuum oven at 60 °C for 12 h to a constant weight.

NMR spectroscopy

NMR spectra were recorded on a Varian Inova or Bruker AV-III 400 MHz spectrometer (400 MHz, ^{1}H; 100 MHz, ^{13}C). Chemical shifts for ^{1}H and ^{13}C spectra were referenced to internal solvent resonance 7.26 (chloroform) and are reported as parts per million relative to $SiMe_4$. Signals are reported as integration, multiplicity (s = singlet, d = doublet, t = triplet, q = quartet, m = multiplet or unresolved, br = broad signal), coupling constant(s) in Hz, assignment. Triad [*mm*], [*rr*], and [*rm*] + [*mr*] values of P3HB were calculated according to the integration area of *mm, rr*, and *mr* + *rm* triads [A(*mm*), A(*rr*), A(*mr* + *rm*)] of the methylene group region, which is calculated as [*rr*] = A(*rr*)/[A(*mm*) + A(*rr*) + A(*mr* + *rm*)] and so on.

Size-exclusion chromatography

The size exclusion chromatography (SEC) instrument employed to study all but two samples ($P3HB_{[29/40/31]}$ and $P3HB_{[33/41/26]}$) is an Agilent HPLC system equipped with one guard column and two PLgel 5 µm mixed-C gel permeation columns coupled with a Wyatt DAWN HELEOS II multi (18)-angle light scattering detector and a Wyatt Optilab TrEX dRI detector. Analysis was performed at 40 °C using $CHCl_3$ as the eluent at a flow rate of 1.0 mL/min, using Wyatt ASTRA 8.2.0.242 molecular weight characterization software. Samples $P3HB_{[29/40/31]}$ and $P3HB_{[33/41/26]}$ were characterized using an Agilent HPLC system equipped with one guard column and two PLgel 5 µm mixed-C gel permeation columns coupled with a Wyatt DAWN HELEOS II multi (18)-angle light scattering detector and a Wyatt Optilab TrEX dRI detector. Analysis was performed at 40 °C using $CHCl_3$ as the eluent at a flow rate of 1.0 mL/min, using Wyatt ASTRA 7.1.2 molecular weight characterization software. All samples were run using the "assume 100 % mass recovery option" which calculated a d*n*/d*c* internally (~0.0254 for *it*-P3HB, ~0.0288 for *sr*-P3HB) based on a precisely known polymer concentration of the sample prior to injection, which were ~2.00 - 4.00 mg/mL.

Differential scanning calorimetry

Differential Scanning Calorimetry (DSC) was performed on an Auto Q20 TA Instrument on polymer samples (2-4 mg) which were dried at ~60 °C for 12 h prior to analysis. DSC plots show the melting-transition temperature (T_m), glass transition temperature (T_g), crystallization temperature (T_c), and enthalpy of fusion (ΔH_f), obtained from a second heating scan after the thermal history was removed on the first heating scan. The first heating scan was performed at a rate of 10 °C/min, the subsequent cooling scan was performed at a rate of 10 °C/min, followed by a second heating scan that was performed at rate of 10 °C/min.

Mechanical testing

Tensile stress/strain testing was performed on an Instron 5966 universal testing system (10 kN load cell) on three dog-bone-shaped test specimens (ASTM D638 standard Type V). Samples $P3HB_{[49/37/14]}$ and $P3HB_{[57/34/9]}$ were tested on an Instron 68TM-10 Mechanical Tester (500 N load cell, 1 kN pneumatic grips) on three dog-bone-shaped specimens (ASTM D638 standard Type V). All specimens were prepared via compression molding using a Carver Auto Series Plus Laboratory Press (Carver, Model 3889.1PL1000, Max Force 15 ton) with programmable electrically heated platen (EHP) temperature, and air-/water-controlled cooling. Isolated polymer materials were loaded between non-stick Teflon paper sheets into a stainless-steel mold with inset dimensions 30 x 73.5 x 0.41 mm fabricated in-house and compressed between the two EHPs at a clamp force of 5000 psi, at temperature ~5 °C higher than each material's respective T_m for 10 min then the EHPs were turned off and the sample was left under pressure until it reached ambient temperature (slow cooling). Specimens for analysis were generated via compression

molding and cut using an ASTM D638-5-IMP cutting die (Qualitest) to standard dimensions. From each compression molding procedure using the stainless-steel mold described, three ASTM D638-5 standard dog-bone shaped specimens could be cut. Mechanical performance was averaged for all the specimens measured for each individual species investigated. Thickness (0.30 ~ 0.50 mm), width (3.18 mm), and grip length (30.0 mm) of the measured dog-bone specimens were measured for normalization of data by the Bluehill measurement software (Instron). Test specimens were affixed into the screw-tight grip frame. Tensile stress and strain were measured to the point of material break at a grip extension speed of 5.0 mm min^{-1} at ambient conditions. Young's modulus (E, MPa), ultimate strength (σ_B, MPa), and elongation at break (ε_B, %) are obtained from the software analysis, Bluehill Universal software (TA). Toughness values (U_T; MJ m^{-3}) were obtained by manual calculation (integration) of the area under the stress/strain curve.

Samples for lap-shear testing were prepared according to a modified version of ASTM D1002. The polymer sample (~30 mg) was placed between two substrates (overlapping area: ~18 mm × 8 mm) in a single lap shear configuration, and then was heated to melt with a heating gun. Upon cooling down to room temperature, the adhesive solidified, and the bond formed between the adhesive layer and the substrate. The specimens were clipped with a binder clip, left in a vacuum oven at 60 °C for 24 h, and then cooled down to room temperature before performing the lap shear test. The samples were affixed into the screw-tight grip frame of an Instron 5966 universal testing system (10 kN load cell) and pulled at a grip extension speed of 5.0 mm min^{-1} at ambient conditions. Adhesive strength (MPa) was calculated by dividing the maximum Force (N) measured by the instrument by the surface area (mm^2) of the applied adhesive.

Peel testing was performed in an Instron 68TM-10 Mechanical Tester (50 N load cell, 50 N pneumatic grips). Samples for peel testing were prepared according to a modified version of ASTM D3330. The adhesive layer was applied to the end of the tape backing strip and adhered on at 50 °C under 1000 PSI pressure for ~1 min between two Teflon sheets using a Carver Auto Series Plus Laboratory Press (Carver, Model 3889.1PL1000, Max Force 15 ton) with programmable electrically heated platen (EHP) temperature, and air-/water-controlled cooling. Once ready for peel testing the tape was removed from the Teflon sheet and applied to the aluminum substrate by hand and tested immediately. Peel strength (N cm^{-1}) was calculated by dividing the maximum Force (N) divided measured by the instrument by the width of the tape backing (mm). Samples were then reported as an average of three tests with error bars showing the standard deviation.

Recycling of Mono-Material Products

Recycling of Tape 1 was carried out according to literature procedures in DCM.[42] The thermal and mechanical properties of the resulting blend were characterized (*vide supra*).

Dynamic Mechanical Analysis (DMA)

Hysteresis experiments were conducted under a preload force of 0.0010 N on a Q800 DMA Analyzer (TA Instruments) in tension film mode.

Rheological Analysis

Rheological measurement was performed using stainless-steel parallel plates (D = 25 mm), with 1 mm gap at 1% of strain on the oscillatory rheometer, Discovery HR20 from TA instrument. For the master curves, dynamic oscillatory frequency sweep was performed in the temperature range of 26 - 180 °C with changing angular frequency from 0.1 rad s^{-1} to 100 rad s^{-1}. The temperature sweep measurement used 10 °C min^{-1} as the ramp rate and 25-180 °C as the temperature range. The viscosity was measured at 150 °C in the range of shear rate of 0.001-1000 Hz with 5 sec of equilibration time. The master curves of $P3HB_{[29/40/31]}$, $P3HB_{[33/41/26]}$, and $P3HB_{[41/39/20]}$ were constructed by using the Williams-Landel-Ferry (WLF) equation. The WLF shift factor, parameters, and each sample's activation energy were derived as follows:

The Williams-Landel-Ferry (WLF) equation:

$$\log a_T = \frac{-c_1(T - T_0)}{c_2 + (T - T_0)} \quad (1)$$

Where a_T is the horizontal shift factor, T_0 is the reference temperature, and c_1 and c_2 are the WLF parameters.

The Arrhenius equation:

$$\log a_T = \frac{E_A}{2.303R}\left(\frac{1}{T} - \frac{1}{T_0}\right) \quad (2)$$

Where R is the universal gas constant (R=8.314 J $mol^{-1}K^{-1}$) and E_A is the activation energy of flow.

Using these equations, the master curves of $P3HB_{[29/40/31]}$, $P3HB_{[33/41/26]}$, and $P3HB_{[41/39/20]}$ were constructed (Figure S1) which show adhesive properties through both the creep region (G''>G', below 0.01Hz) and tack regions (G'>G'', in between 0.01 and 100Hz), which is appropriate for good adhesives. The plateau modulus (G_N^0) can be obtained from the G'(ω) value at the frequency ω, where the minimum of tan δ is located, which represents the maximum elasticity of the system. The plateau modulus also relates to the entanglement molecular weight (M_e) as the following equation.[55]

$$G_N^0 = G'(w)_{tan\delta_{min}} = \frac{\rho RT}{M_e} \tag{3}$$

The density of the polymer melts (ρ = 1.06 g ml^{-1}) is estimated from the literature value[56] and the temperature (T) is 303K. The calculated plateau modulus (G_N^0) and the entanglement molecular weight (M_e) (Table S2) and those values are in the same order of magnitude of microbial synthesized poly(hydroxyalkanoate)s.[57]

Furthermore, the shear thinning behavior can be quantitatively analyzed with the Carreau model (Table S3) which is one of the empirical equations used to fit non-Newtonian, pseudoplastic flow such as polymer melts.[58] The effect of mixing ratio on viscosity can be analyzed by the obtained values from this model. The zero-shear viscosity (η_0) is higher in higher meso content, whereas the characteristic relaxation time (k) decreases with increasing meso content. The power law index decreases with increasing meso content, indicating a more rapid drop in viscosity.

The Carreau model:

$$\frac{\eta - \eta_\infty}{\eta_0 - \eta_\infty} = \frac{1}{\left(1 + (k\dot{\gamma})^2\right)^{n-1/2}} \tag{4}$$

Where the zero-shear viscosity (η_0), the infinite viscosity (η_∞), the consistency (k, i.e., onset or characteristic relaxation time), and the power law index (n).

Biodegradation Testing

Biodegradability tests in freshwater environment were conducted using ISO 14851 method[59] for freshwater environment. The biodegradability of polymer samples was conducted in triplicate using 300 mL biological oxygen demand (BOD) glass bottles (VWR International). In each BOD bottle, activated sludge from a wastewater treatment plant (Lemont, IL, USA) was mixed with 200 mL of aqueous medium comprising: KH_2PO_4, 85 mg/mL; K_2HPO_4, 217.5 mg/mL; Na_2HPO_4, 334 mg/mL; NH_4Cl, 15 mg/mL; $MgSO_4 \cdot 7H_2O$, 22.5 mg/mL; $CaCl_2 \cdot 2H_2O$, 36.4 mg/mL; and $FeCl_3 \cdot 6H_2O$, 0.25 mg/mL. Total solid concentration of sludge sample was 60 mg/L. For each of the triplicate test bottles of polymer samples, P3HB sample was added to the BOD bottles. Total organic carbon content of P3HB samples added to every test bottle was 9.0 mg. Three blank test bottles with no additional carbon content other than the aqueous medium and three positive control bottles with 9.0 mg total organic carbon content from either *D*-glucose (Fisher Scientific, granular powder) or cellulose (microcrystalline, particle size 0.05 mm, Acros Organic) were also tested. All these bottles were incubated in a New Brunswick Scientific incubator shaker (Eppendorf, S7 model I-24) at 25 °C and 150 rpm for 90 days. BOD values were determined by measuring oxygen consumption using Thermo Scientific pH/RDO/DO meter (Thermo Fisher Scientific, model Orion Star A216) based on ISO 14851 standard method.

The biodegradability of the sample in percentage was calculated as

$$\%\,Biodegradation = \frac{BOD_{sample} - BOD_{blank}}{c \times ThOD} \times 100\% \tag{5}$$

BOD_{sample} and BOD_{blank} were the observed values of the sample and blank bioreactor, respectively, c was the mass of sample added (mg), and ThOD was the theoretical oxygen demand value of the sample, calculated from the chemical formula and based on the assumption of complete oxidation of polymer sample. The protocol required the positive control (cellulose) to reach 60% biodegradation at the end of the test and the standard deviation of each sample to be less than 20% of the mean.

End of lifetime estimation of polymer samples

The first order kinetic model was used to calculate the biodegradation rate and estimate the lifetime of the polymer samples in freshwater and soil environment.

$$\% \, Biodegradation = 1 - e^{(-kt+C)} \quad (6)$$

Equation 6 was used to plot the percentage biodegradation with respect to time. From the equation, k was the rate constant, t was reaction time, and C was a constant from the plot. Table S13 shows a summary of biodegradation rates constant and estimated times to reach 90% biodegradation in freshwater environment and R^2 values showing the fitness of the model.

Supplementary Figures

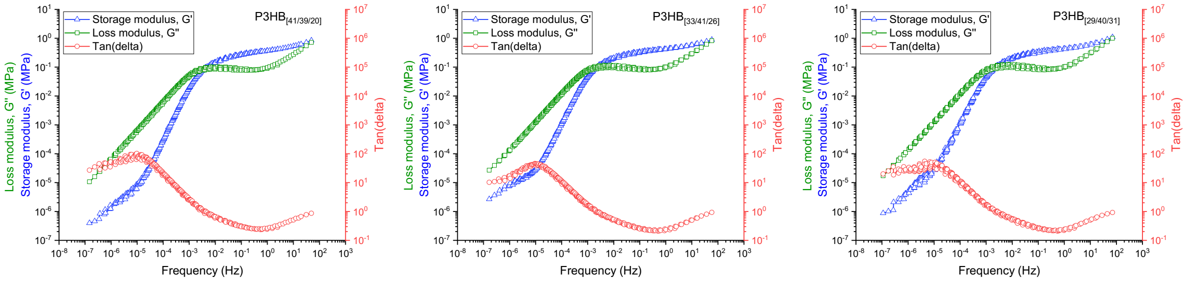

Figure S1. Time-temperature superposition master curves of P3HB polymers.

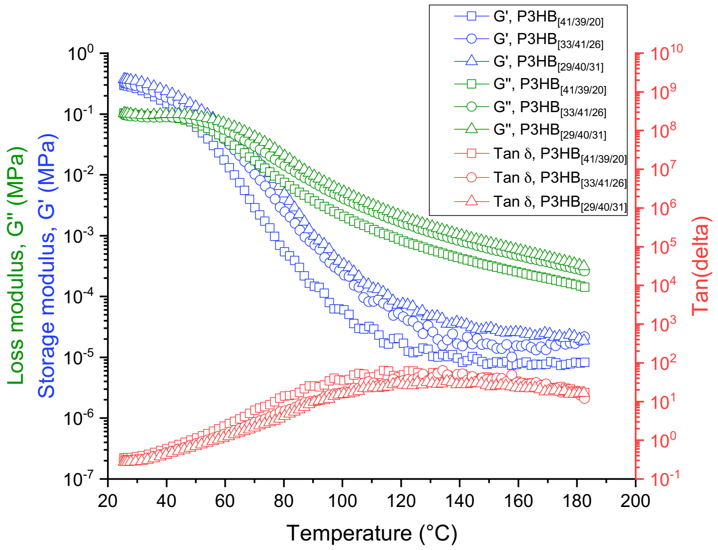

Figure S2. Temperature sweep measurements of P3HB polymers.

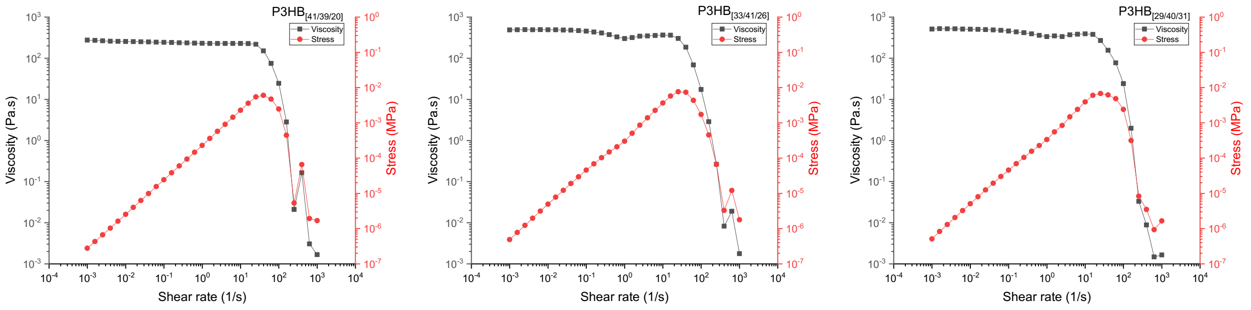

Figure S3. Steady-state shear viscosity of P3HB polymers.

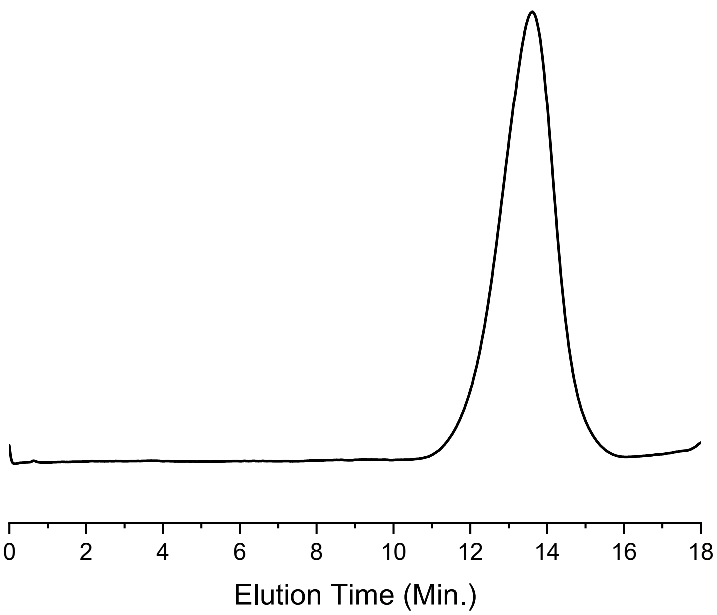

Figure S4. SEC trace of $P3HB_{[0/41/59]}$ (M_n = 216 kg mol^{-1}, M_w = 277 kg mol^{-1}, $Đ$ = 1.29).

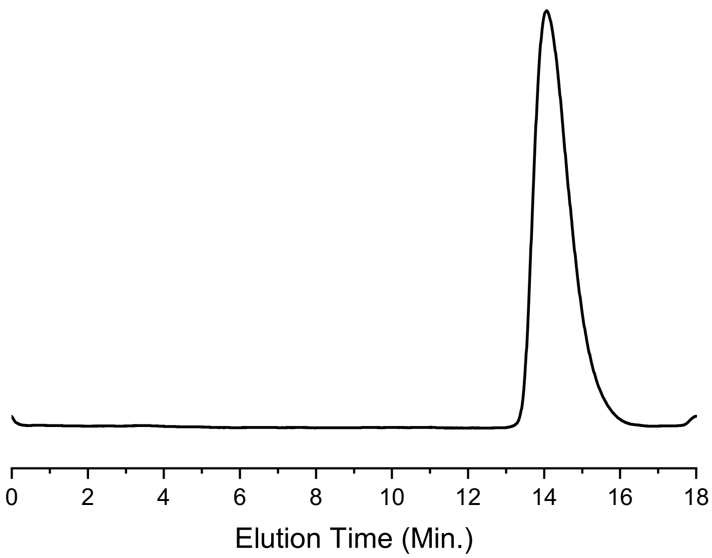

Figure S5. SEC trace of $P3HB_{[4/39/57]}$ (M_n = 142 kg mol^{-1}, M_w = 165 kg mol^{-1}, $Đ$ = 1.16).

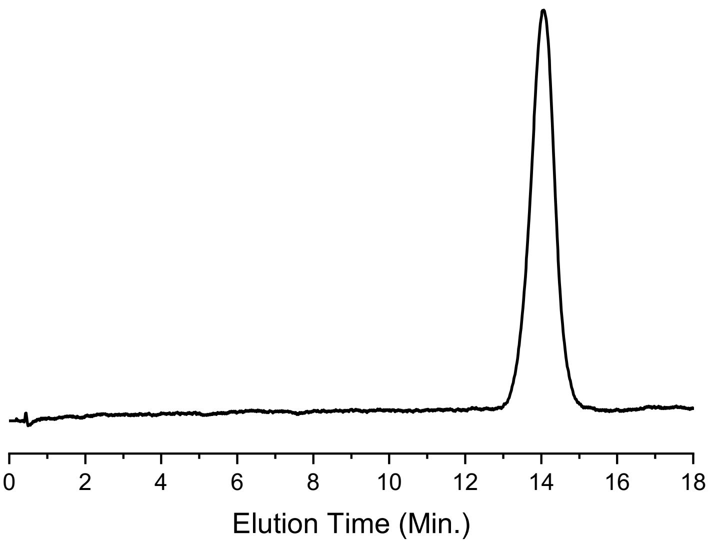

Figure S6. SEC trace of $P3HB_{[10/45/45]}$ (M_n = 126 kg mol^{-1}, M_w = 126 kg mol^{-1}, $Đ$ = 1.01).

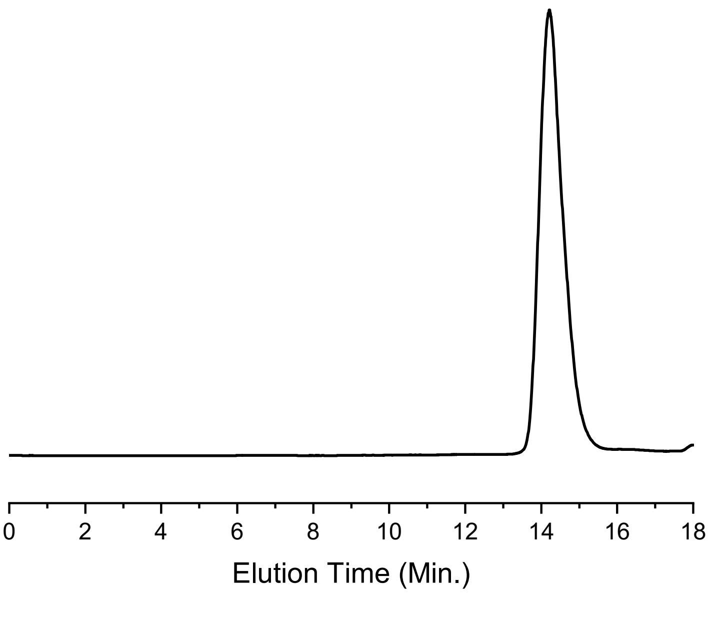

Figure S7. SEC trace of $P3HB_{[18/42/40]}$ (M_n = 111 kg mol^{-1}, M_w = 113 kg mol^{-1}, $Đ$ = 1.01).

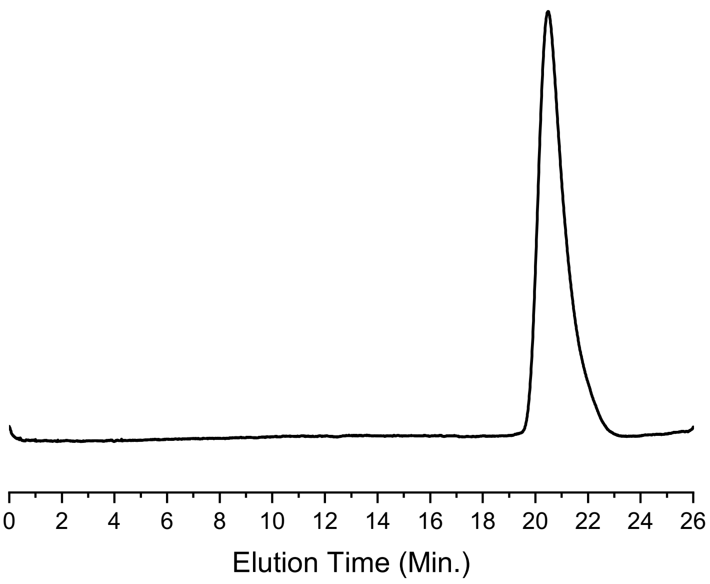

Figure S8. SEC trace of $P3HB_{[29/40/31]}$ (M_n = 105 kg mol^{-1}, M_w = 122 kg mol^{-1}, $Đ$ = 1.16).

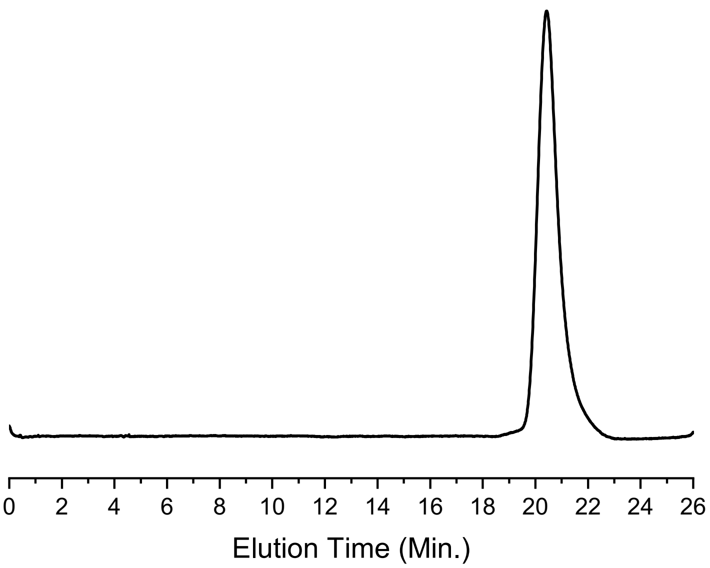

Figure S9. SEC trace of $P3HB_{[33/41/26]}$ (M_n = 144 kg mol^{-1}, M_w = 157 kg mol^{-1}, $Đ$ = 1.10).

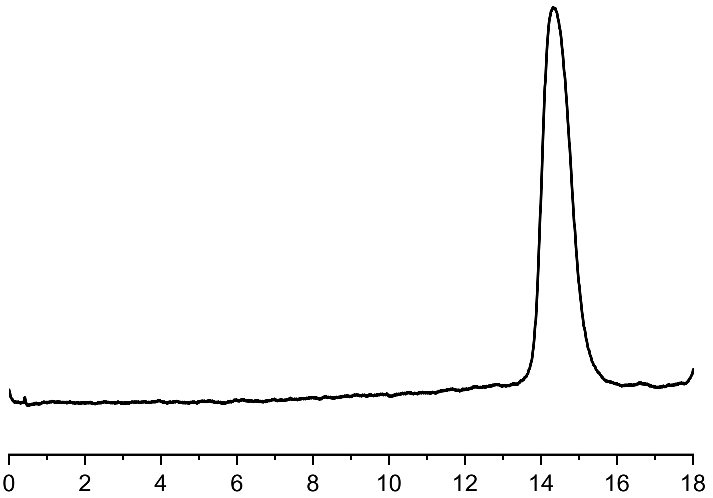

Figure S10. SEC trace of $P3HB_{[41/39/20]}$ (M_n = 105 kg mol^{-1}, M_w = 111 kg mol^{-1}, $Đ$ = 1.06).

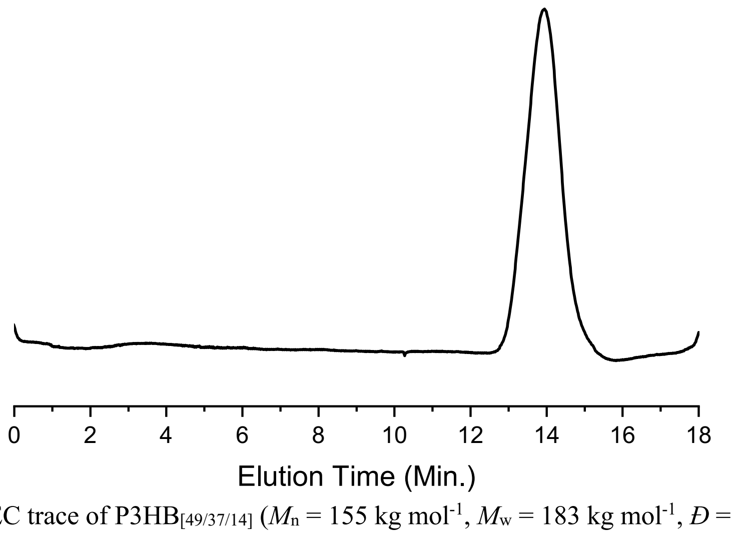

Figure S11. SEC trace of $P3HB_{[49/37/14]}$ (M_n = 155 kg mol^{-1}, M_w = 183 kg mol^{-1}, $Đ$ = 1.18).

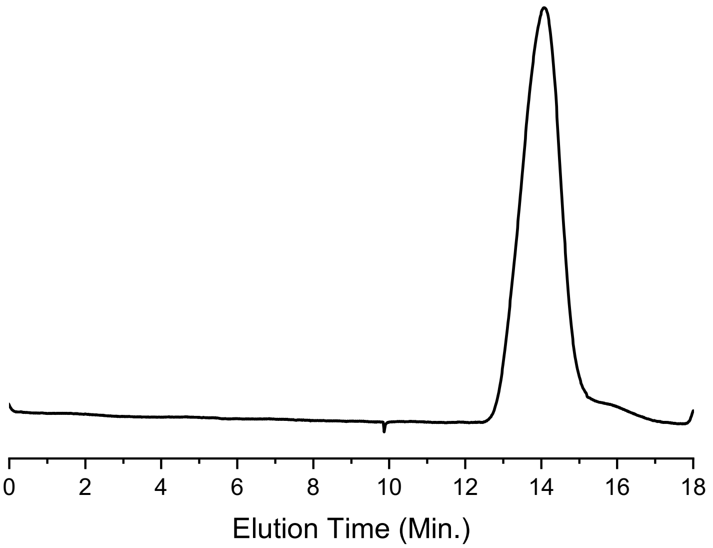

Figure S12. SEC trace of $P3HB_{[57/34/9]}$ (M_n = 195 kg mol^{-1}, M_w = 236 kg mol^{-1}, $Đ$ = 1.21).

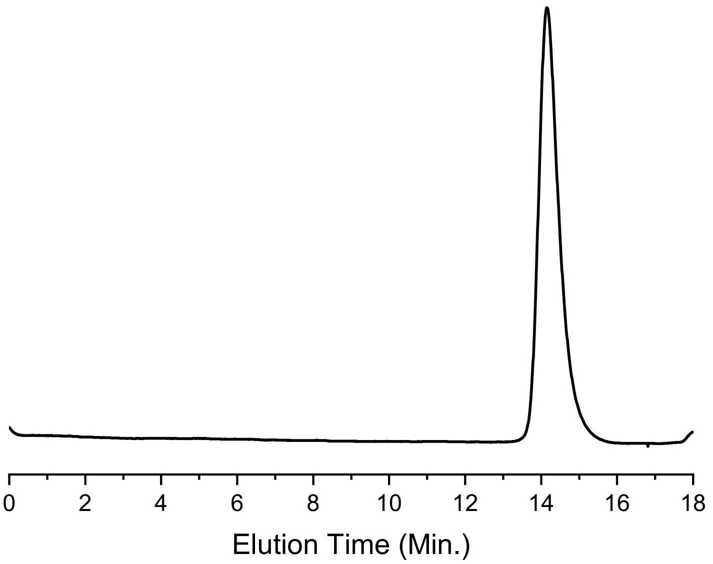

Figure S13. SEC trace of $P3HB_{[63/32/5]}$ (M_n = 102 kg mol^{-1}, M_w = 105 kg mol^{-1}, $Đ$ = 1.03).

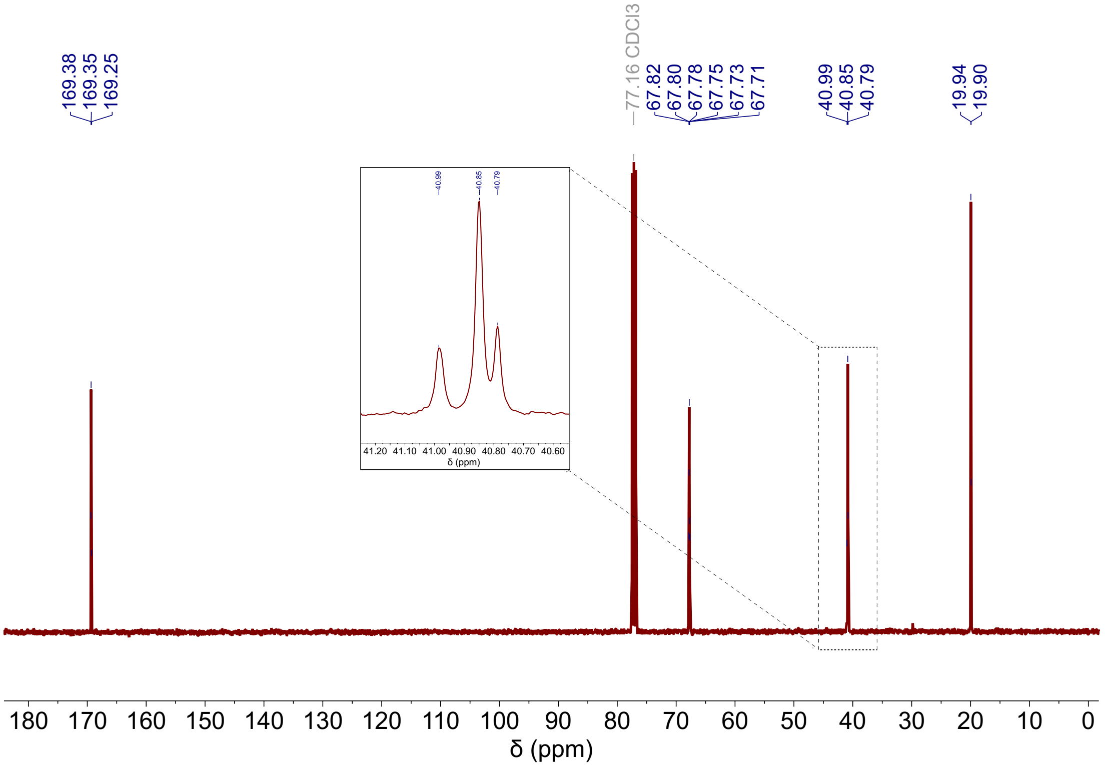

Figure S14. ^{13}C NMR Spectrum ($CDCl_3$, 23 °C) of $P3HB_{[0/41/59]}$ with inset of methylene region.

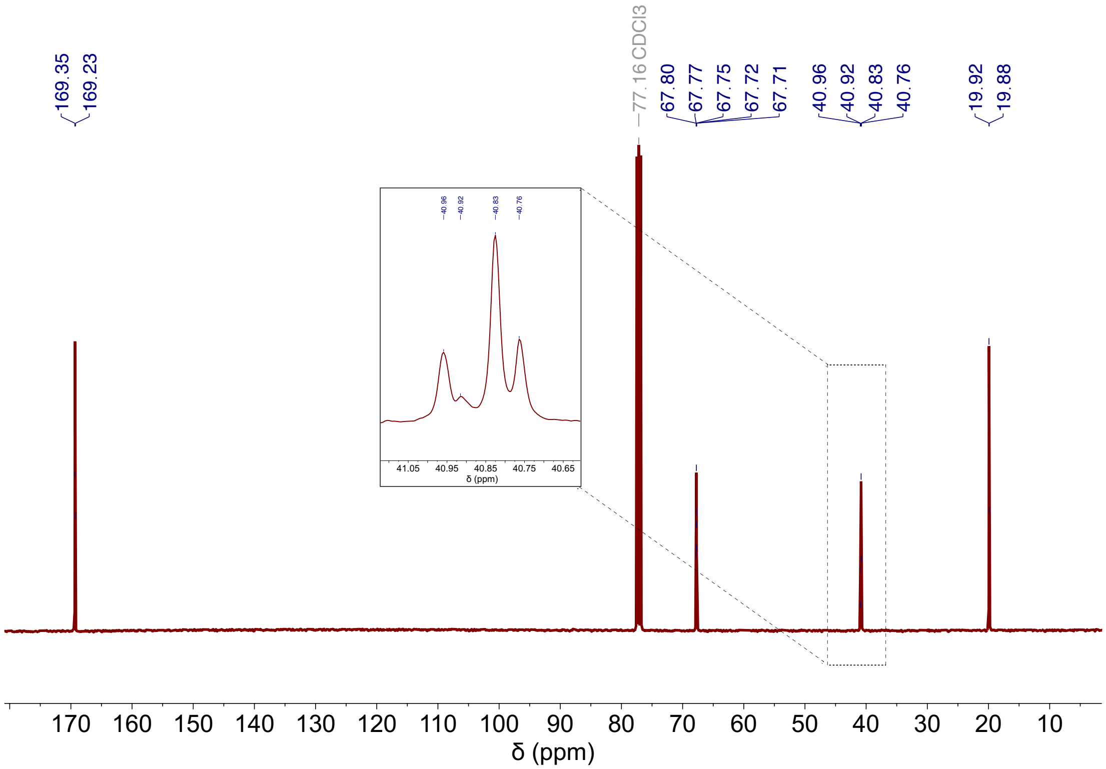

Figure S15. ^{13}C NMR Spectrum ($CDCl_3$, 23 °C) of $P3HB_{[4/39/57]}$ with inset of methylene region.

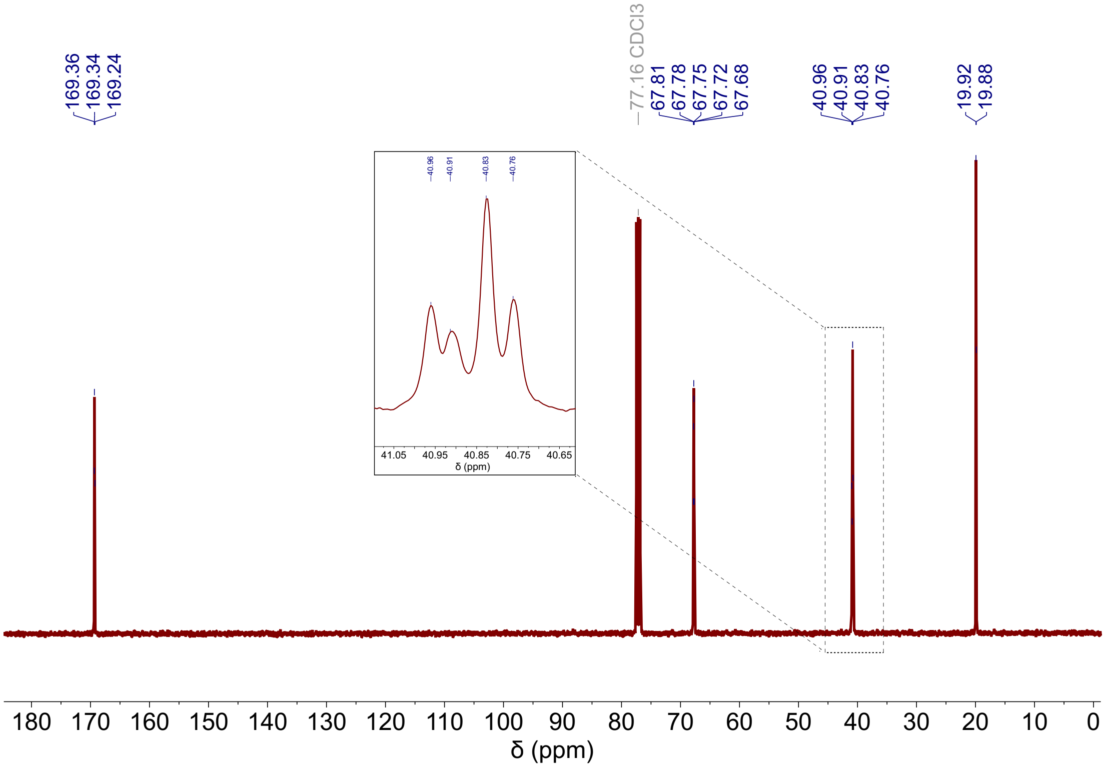

Figure S16. ^{13}C NMR Spectrum ($CDCl_3$, 23 °C) of $P3HB_{[10/45/45]}$ with inset of methylene region.

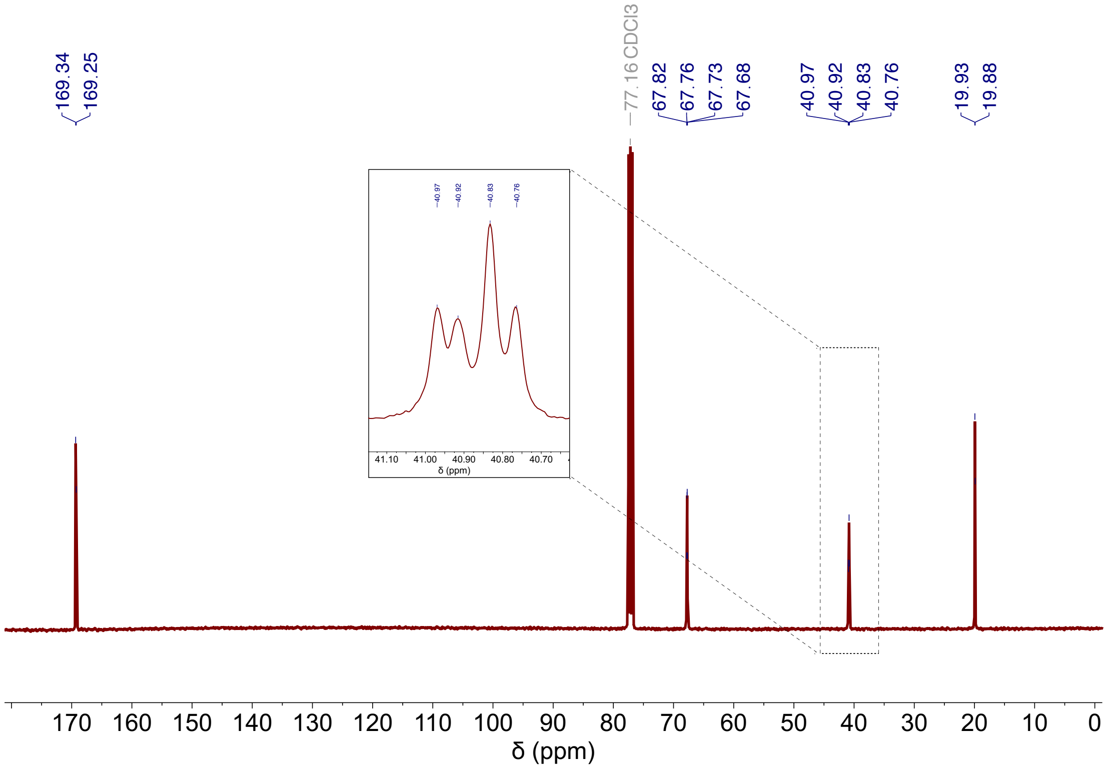

Figure S17. ^{13}C NMR Spectrum ($CDCl_3$, 23 °C) of $P3HB_{[18/42/40]}$ with inset of methylene region.

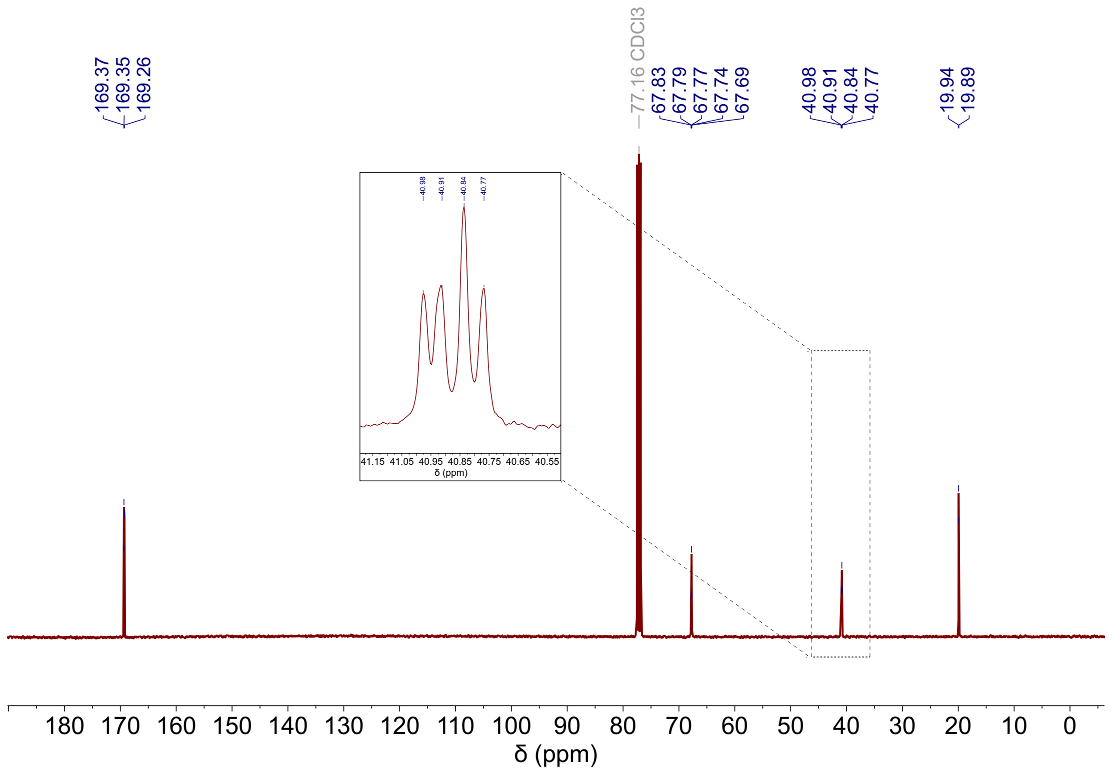

Figure S18. ^{13}C NMR Spectrum ($CDCl_3$, 23 °C) of P3HB$_{[29/40/31]}$ with inset of methylene region.

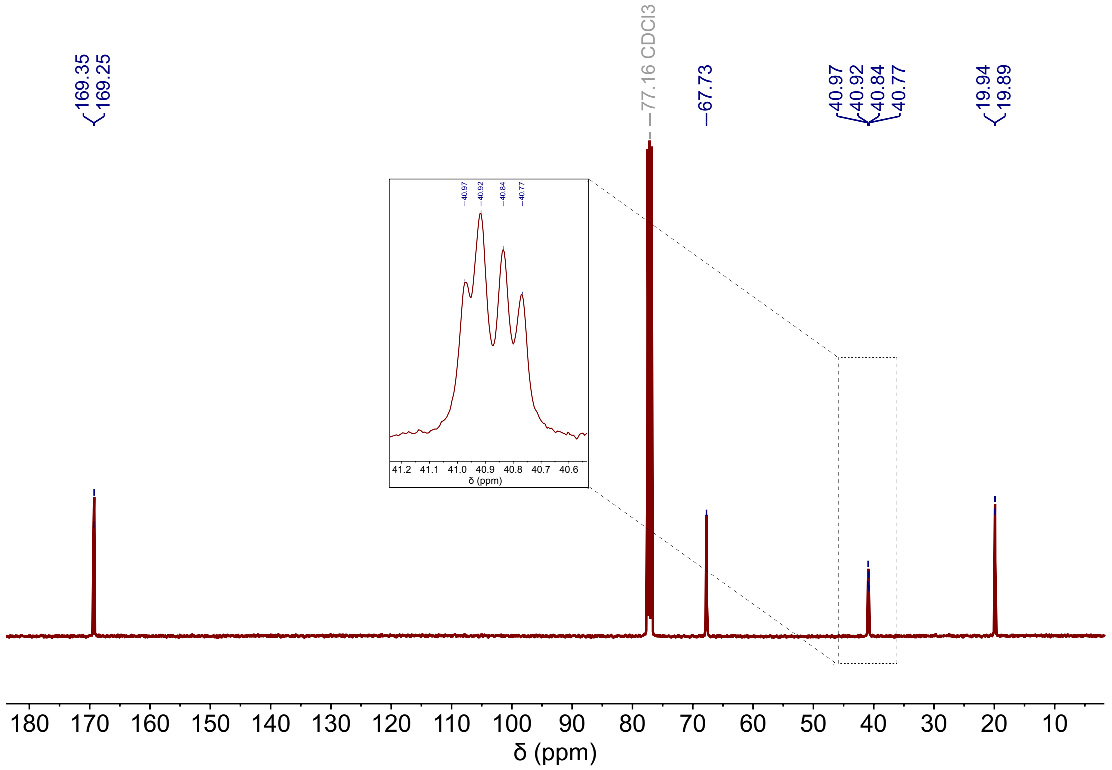

Figure S19. ^{13}C NMR Spectrum ($CDCl_3$, 23 °C) of P3HB$_{[33/41/26]}$ with inset of methylene region.

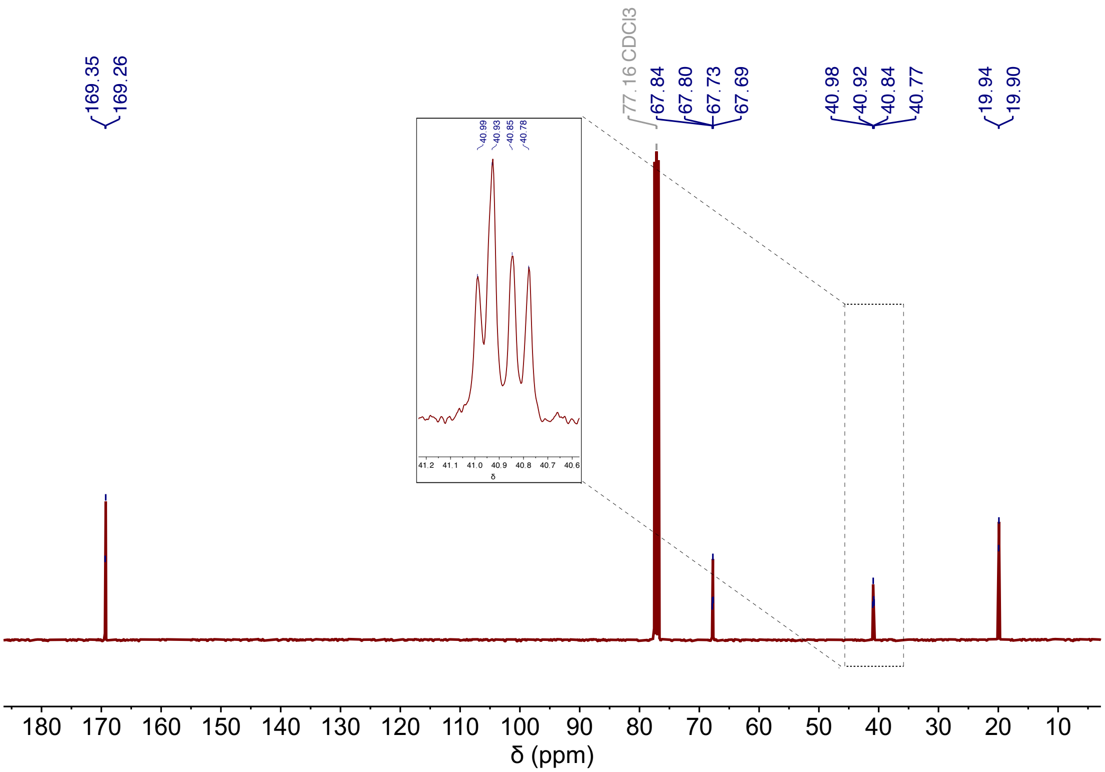

Figure S20. ^{13}C NMR Spectrum ($CDCl_3$, 23 °C) of $P3HB_{[41/39/20]}$ with inset of methylene region.

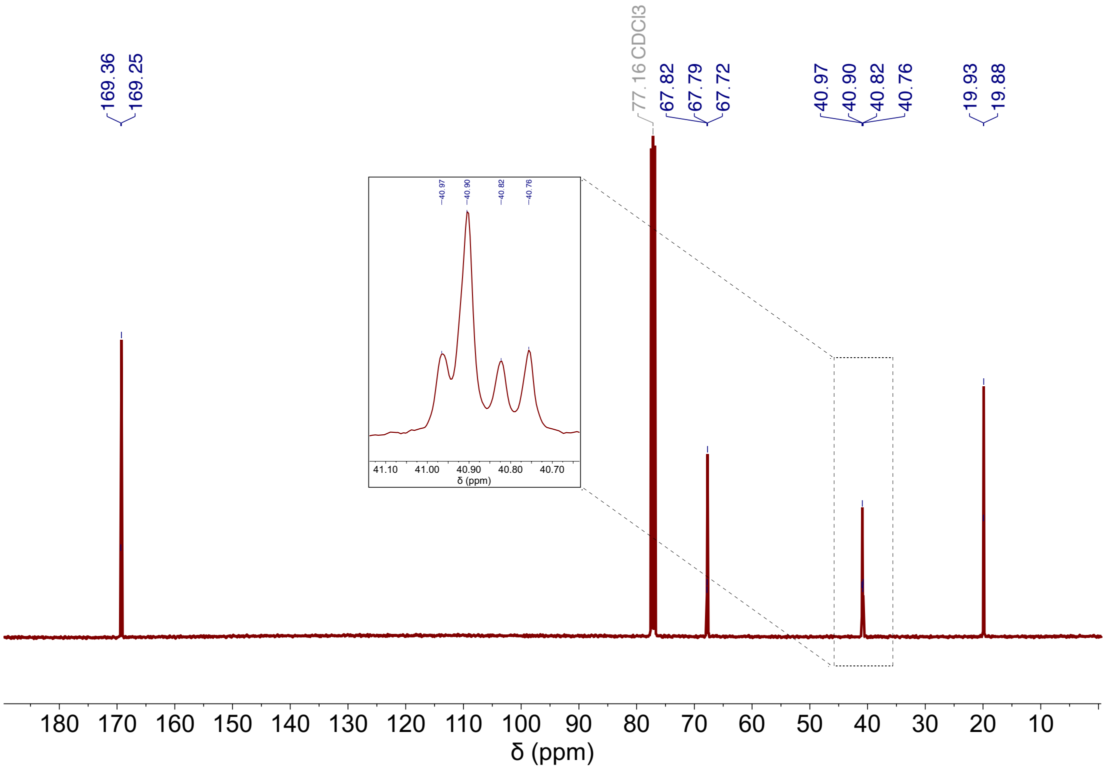

Figure S21. ^{13}C NMR Spectrum ($CDCl_3$, 23 °C) of $P3HB_{[49/37/14]}$ with inset of methylene region.

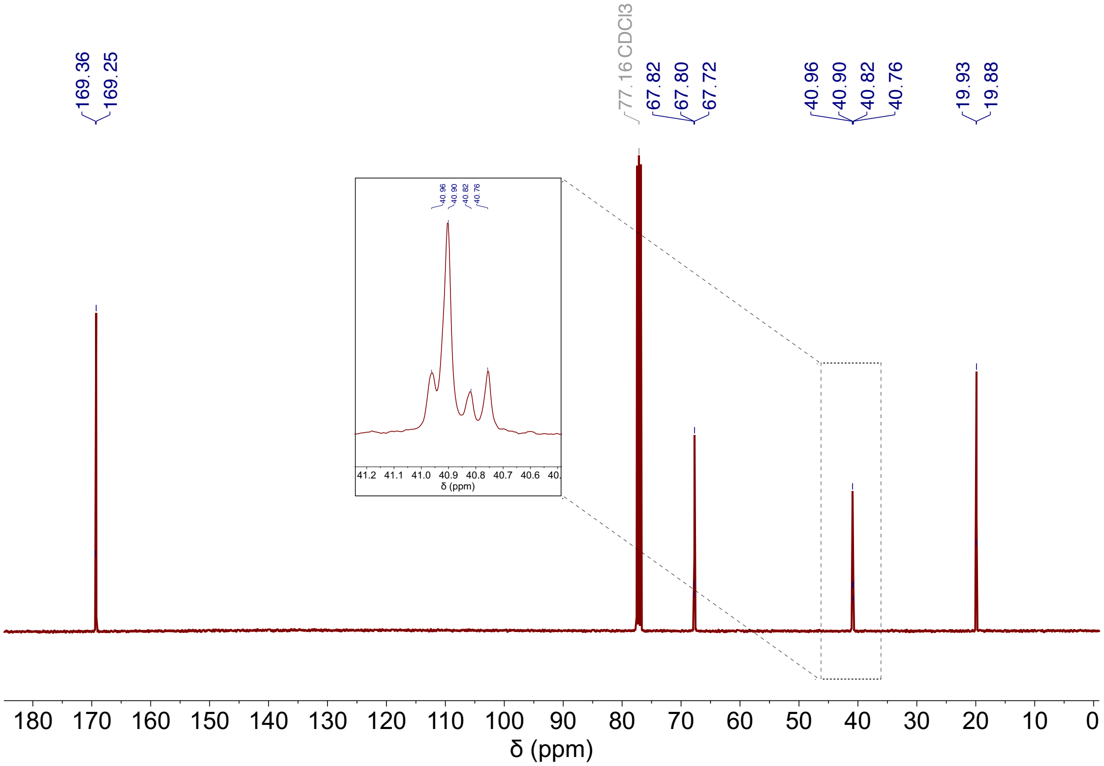

Figure S22. ^{13}C NMR Spectrum ($CDCl_3$, 23 °C) of $P3HB_{[57/34/9]}$ with inset of methylene region.

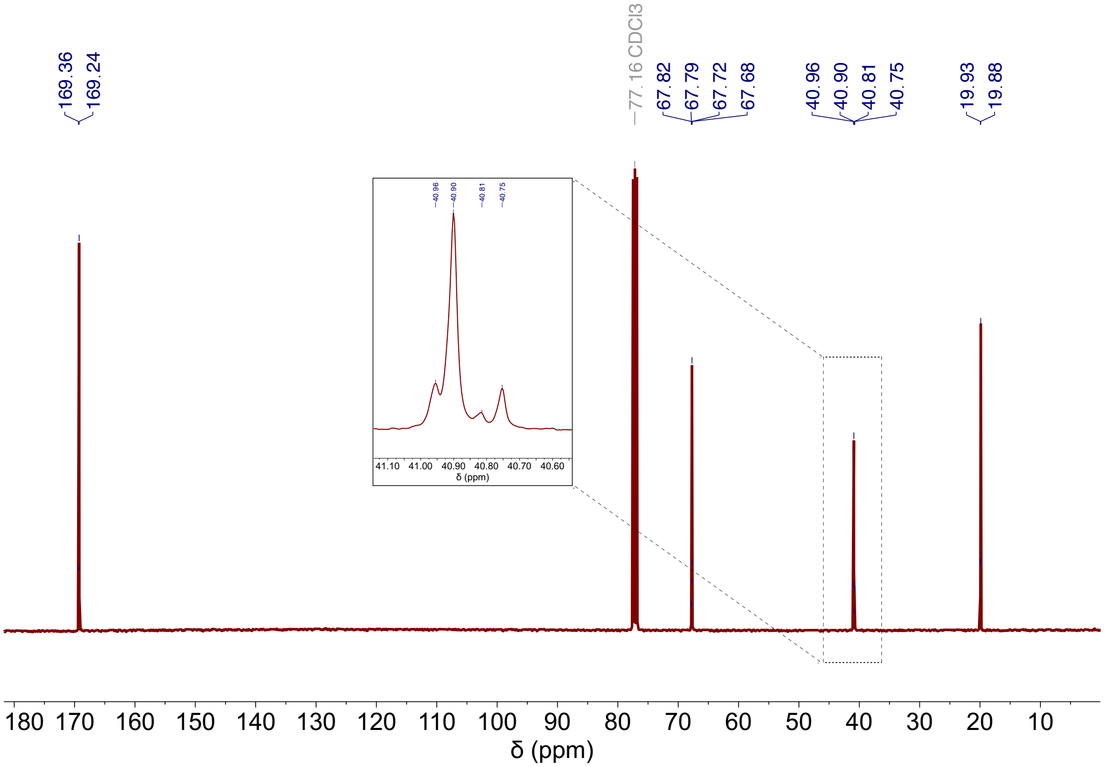

Figure S23. ^{13}C NMR Spectrum ($CDCl_3$, 23 °C) of $P3HB_{[63/32/5]}$ with inset of methylene region.

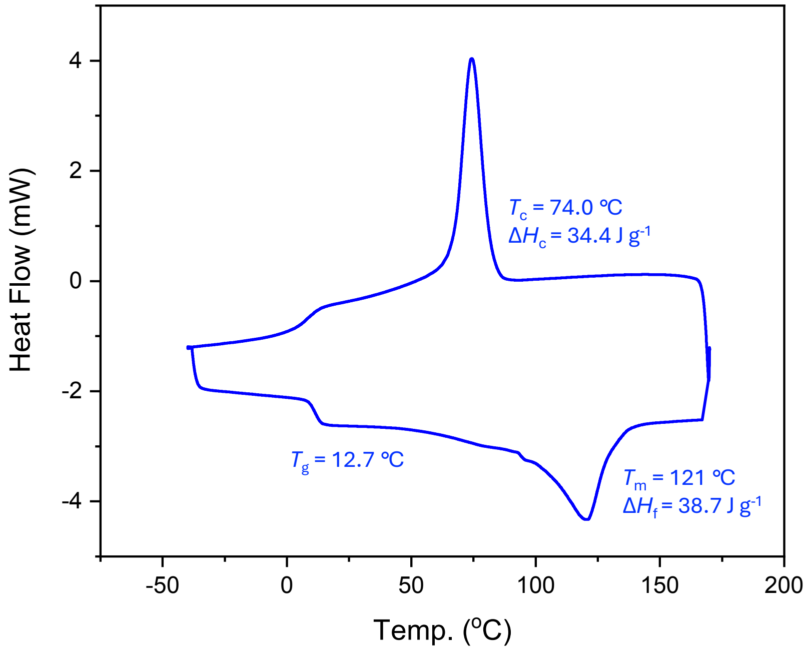

Figure S24. DSC curve of P3HB$_{[0/41/59]}$ (M_n = 216 kg mol^{-1}, $Đ$ = 1.29). 2nd heating scan and cooling scan.

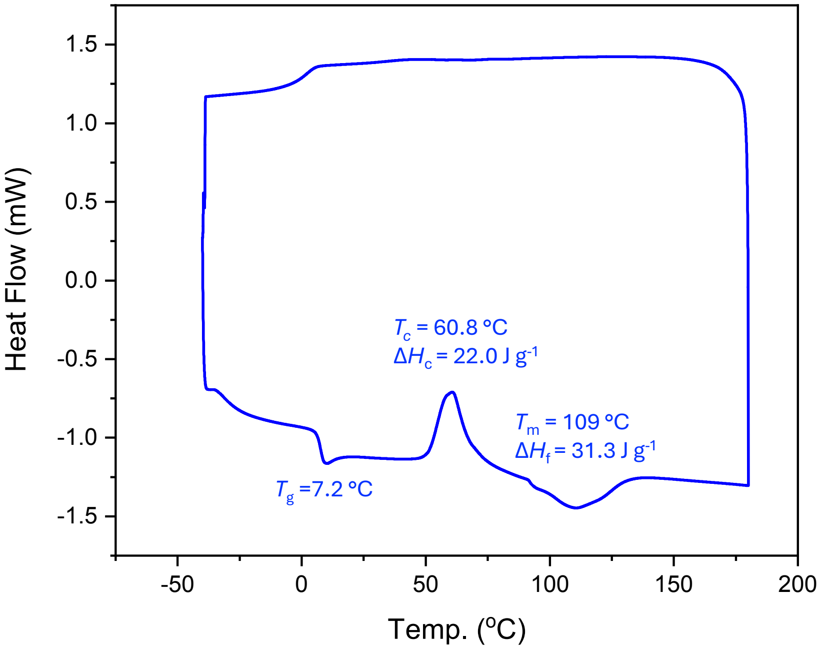

Figure S25. DSC curve of P3HB$_{[4/39/57]}$ (M_n = 142 kg mol^{-1}, $Đ$ = 1.16). 2nd heating scan and cooling scan.

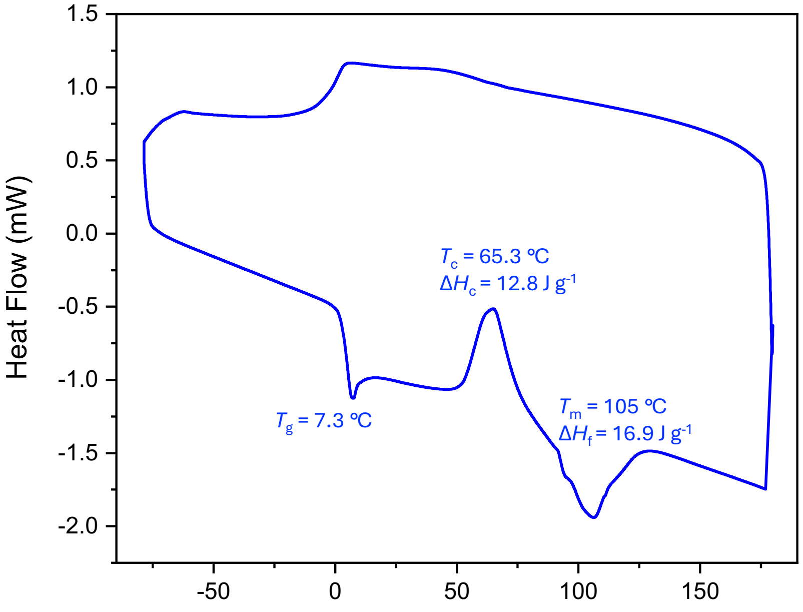

Figure S26. DSC curve of P3HB$_{[10/45/45]}$ (M_n = 126 kg mol^{-1}, $Đ$ = 1.01). 2nd Heating scan and cooling scan.

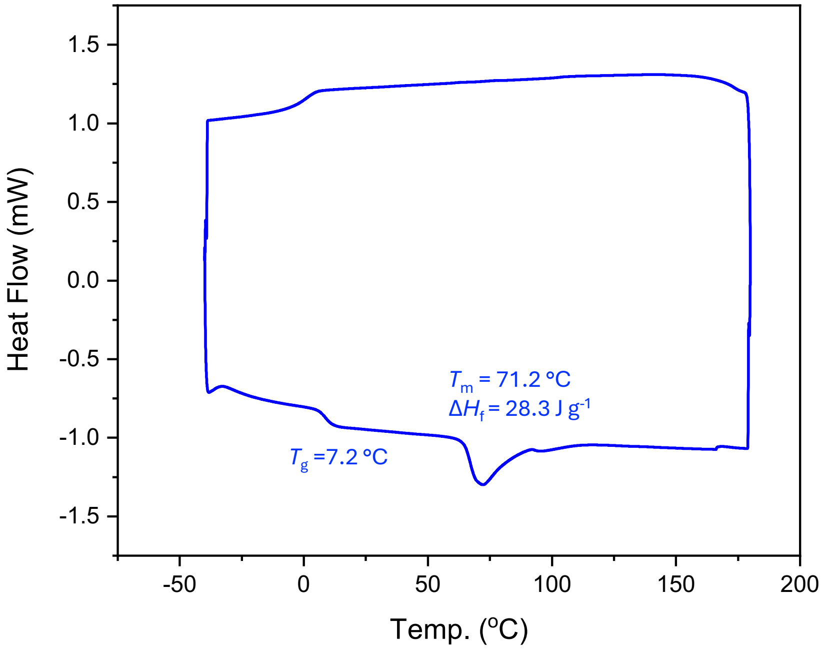

Figure S27. DSC curve of P3HB$_{[18/42/40]}$ (M_n = 111 kg mol^{-1}, $Đ$ = 1.01). 1st heating scan and cooling scan.

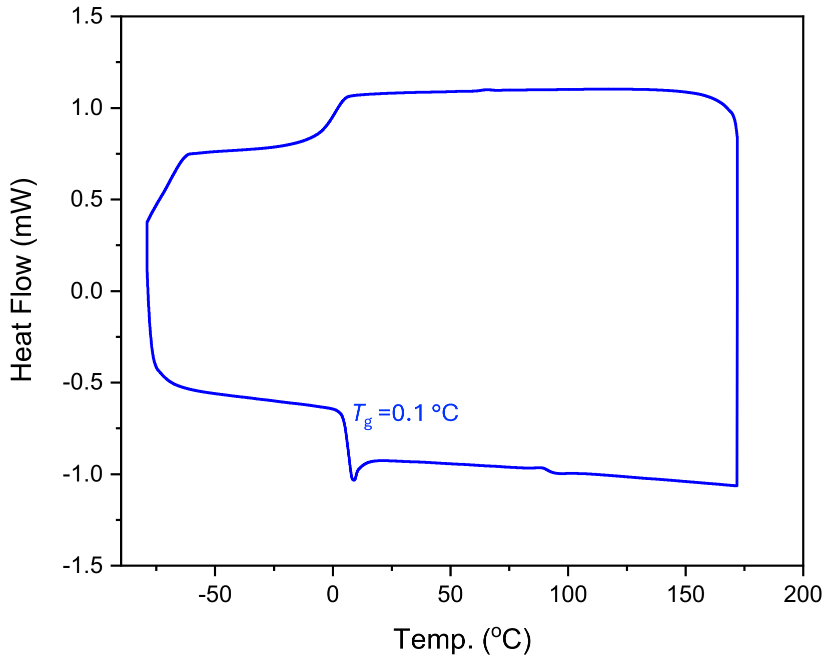

Figure S28. DSC curve of P3HB$_{[29/40/31]}$ (M_n = 105 kg mol^{-1}, $Đ$ = 1.16). 1st heating scan and cooling scan.

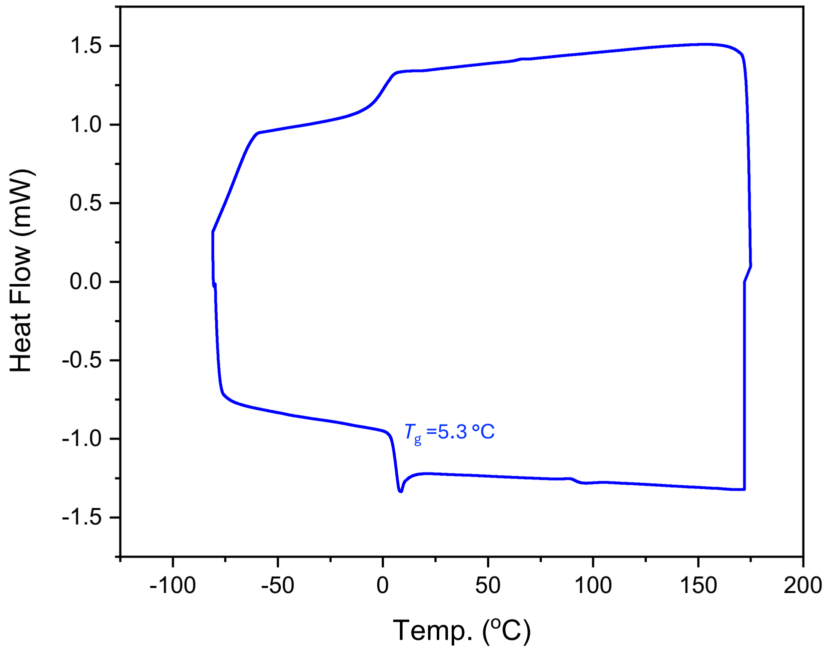

Figure S29. DSC curve of P3HB$_{[33/41/26]}$ (M_n = 144 kg mol-1, $Đ$ = 1.10). 1st heating scan and cooling scan.

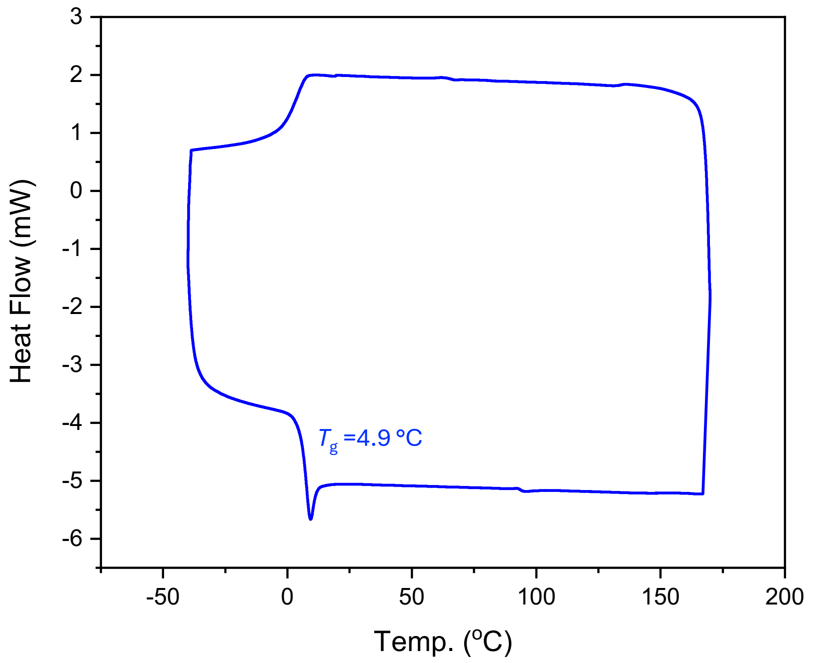

Figure S30. DSC curve of P3HB$_{[41/39/20]}$ (M_n = 105 kg mol^{-1}, $Đ$ = 1.06). 1st heating scan and cooling scan.

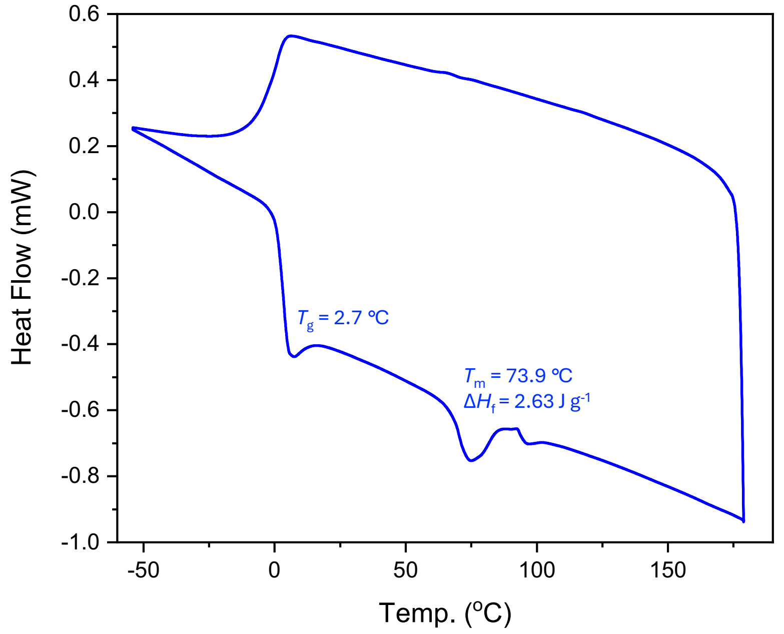

Figure S31. DSC curve of P3HB$_{[49/37/14]}$ (M_n = 155 kg mol^{-1}, $Đ$ = 1.18). 1st heating scan and cooling scan. DSC artifact observed from 92-100 °C.

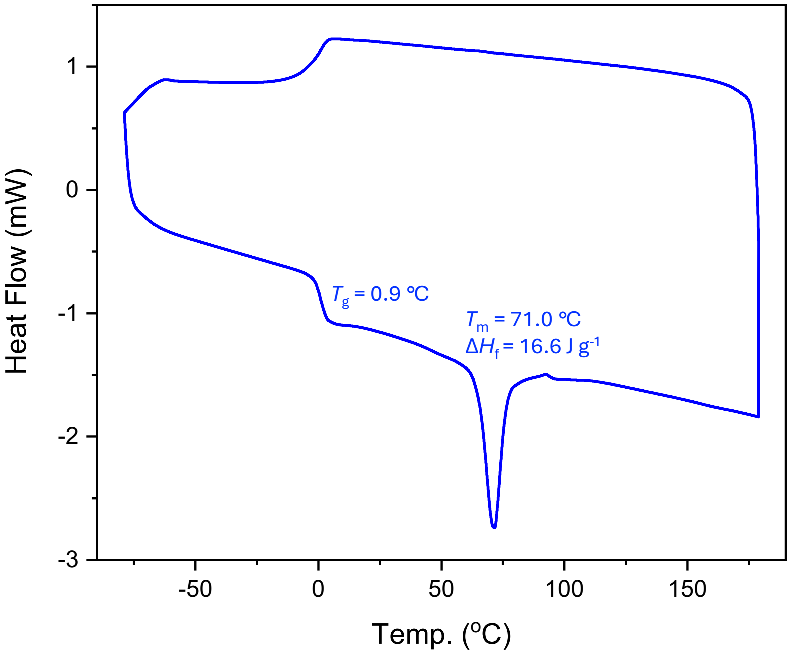

Figure S32. DSC curve of P3HB$_{[57/34/9]}$ (M_n = 195 kg mol^{-1}, $Đ$ = 1.21). 1st heating scan and cooling scan. DSC artifact observed from 92-97 °C.

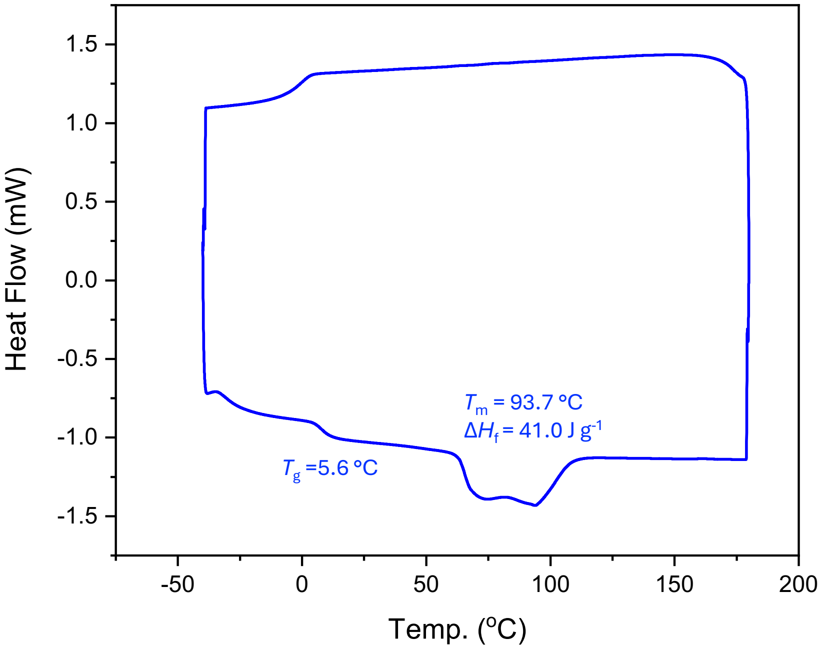

Figure S33. DSC curve of P3HB$_{[63/32/5]}$ (M_n = 102 kg mol^{-1}, $Đ$ = 1.03). 1st heating scan and cooling scan.

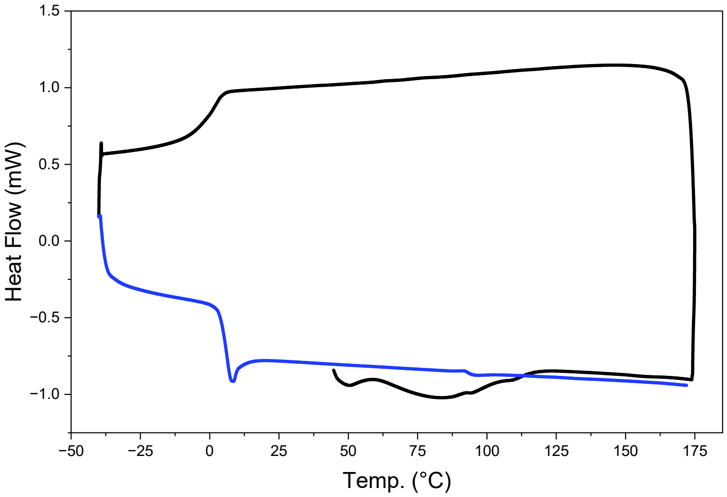

Figure S34. DSC curve of recycled Tape 1. Black line is the 1st heating scan and cooling scan; blue line is the second heating scan.

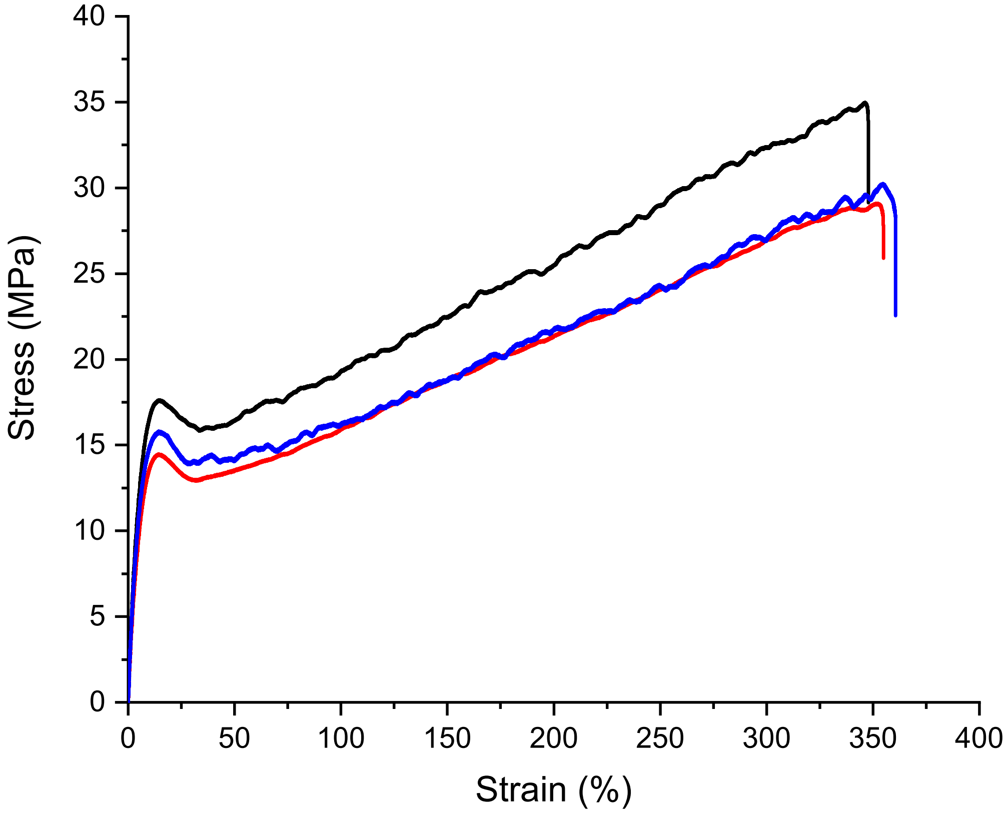

Figure S35. Triplicate stress-strain curves of $P3HB_{[0/41/59]}$ (M_n = 216 kg mol^{-1}, $Đ$ = 1.29).

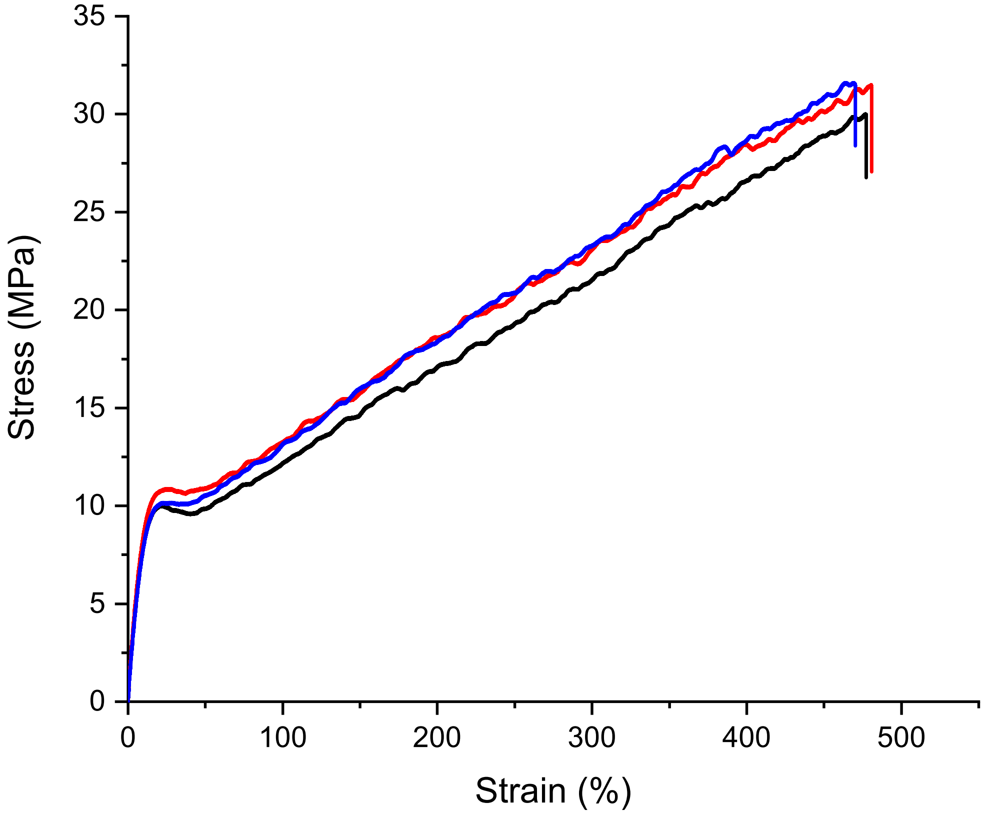

Figure S36. Triplicate stress-strain curves of $P3HB_{[4/39/57]}$ (M_n = 142 kg mol^{-1}, $Đ$ = 1.16).

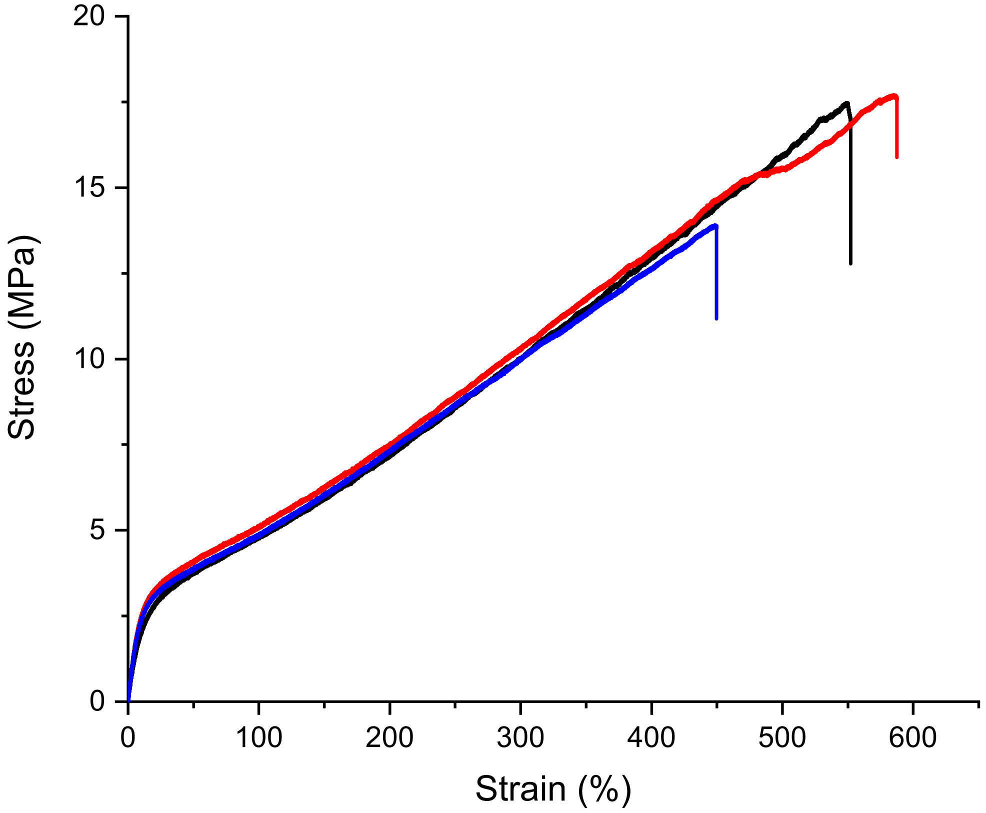

Figure S37. Triplicate stress-strain curves of P3HB[10/45/45] (Mn = 126 kg mol-1, Đ = 1.01).

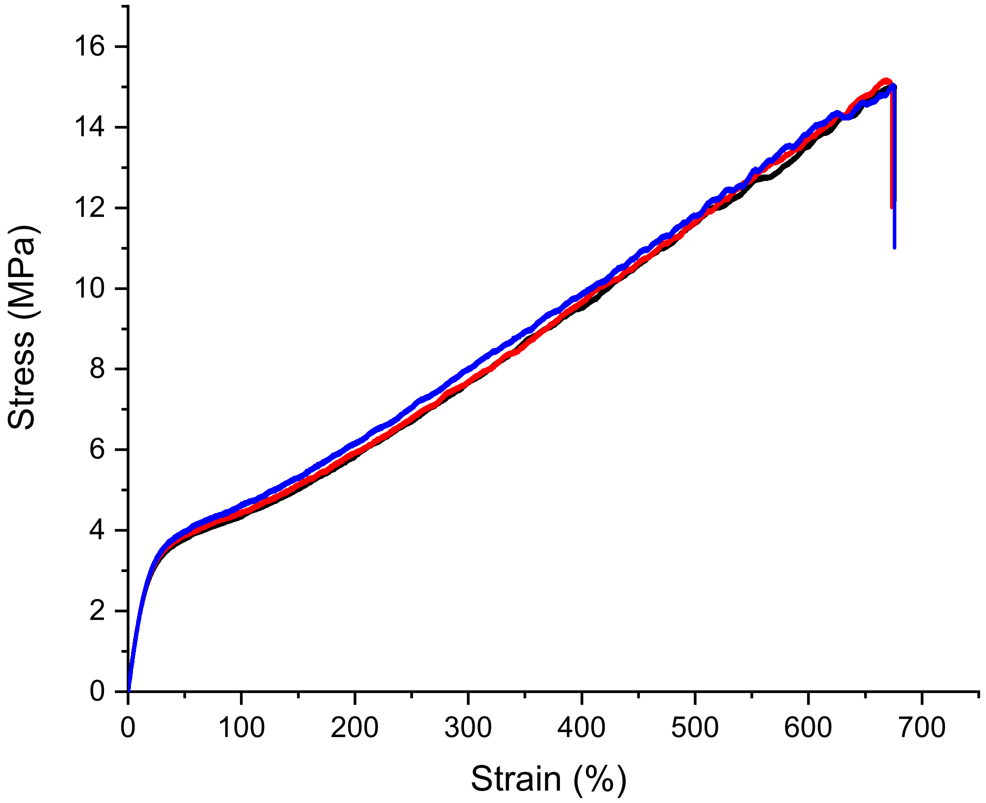

Figure S38. Triplicate stress-strain curves of $P3HB_{[18/42/40]}$ (M_n = 111 kg mol^{-1}, $Đ$ = 1.01).

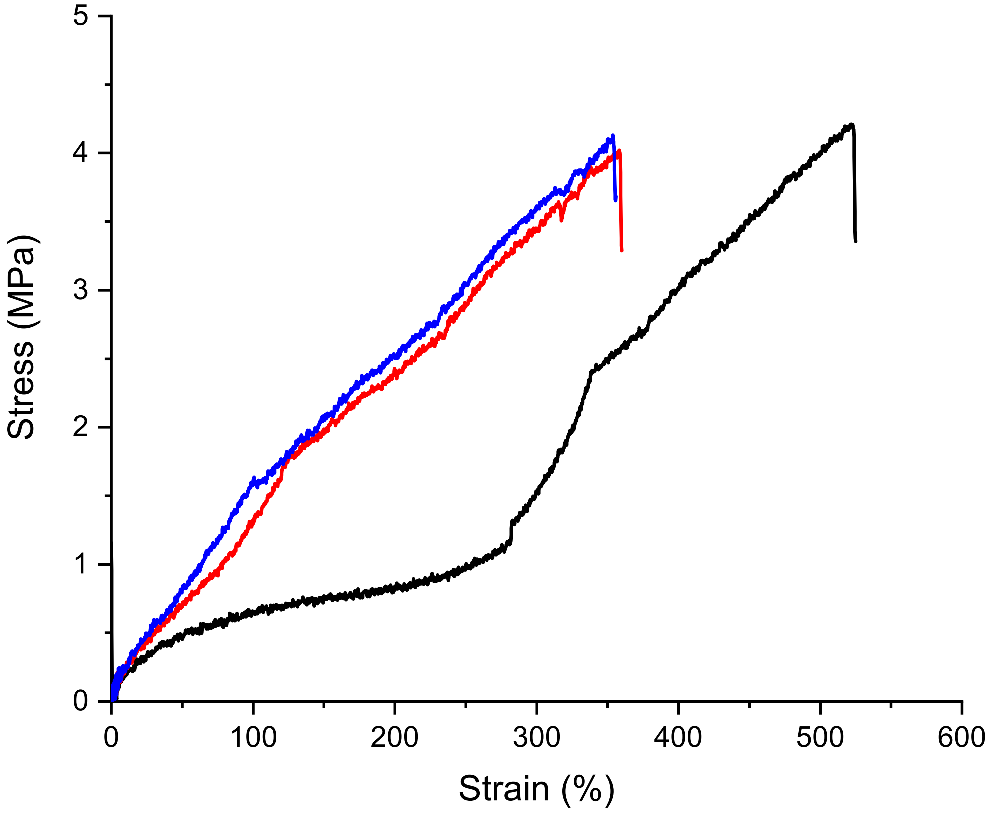

Figure S39. Triplicate stress-strain curves of $P3HB_{[49/37/14]}$ (M_n = 155 kg mol^{-1}, $Đ$ = 1.18).

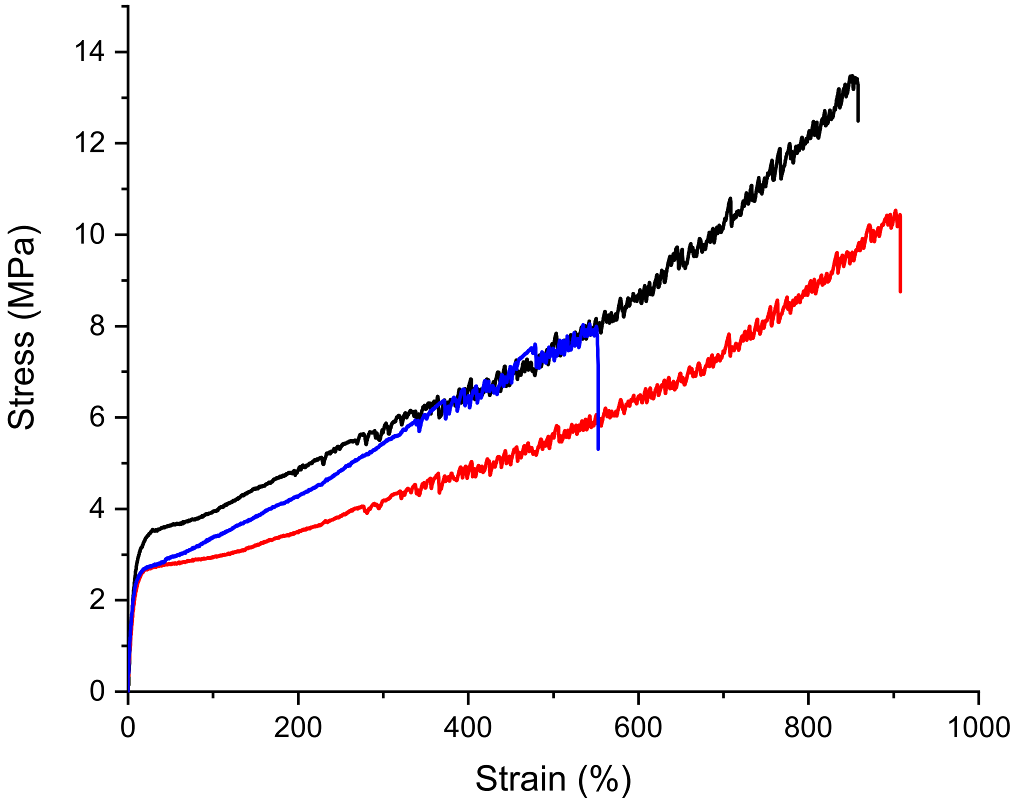

Figure S40. Triplicate stress-strain curves of $P3HB_{[57/34/9]}$ (M_n = 195 kg mol^{-1}, $Đ$ = 1.21).

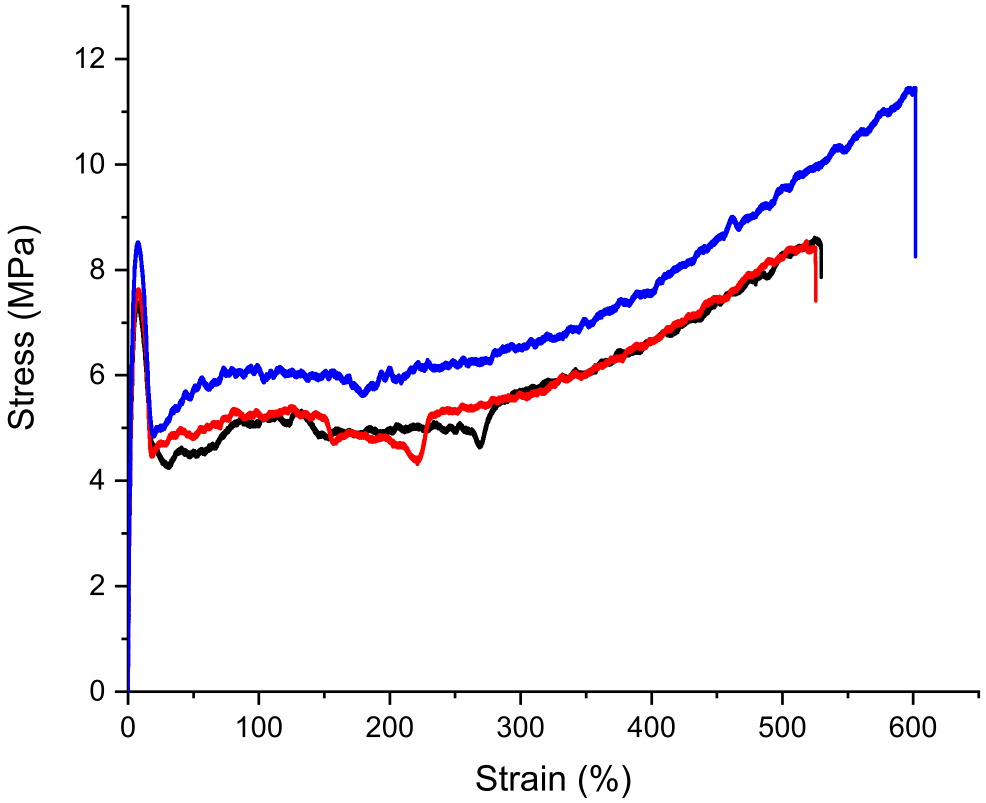

Figure S41. Triplicate stress-strain curves of $P3HB_{[63/32/5]}$ (M_n = 102 kg mol^{-1}, $Đ$ = 1.03).

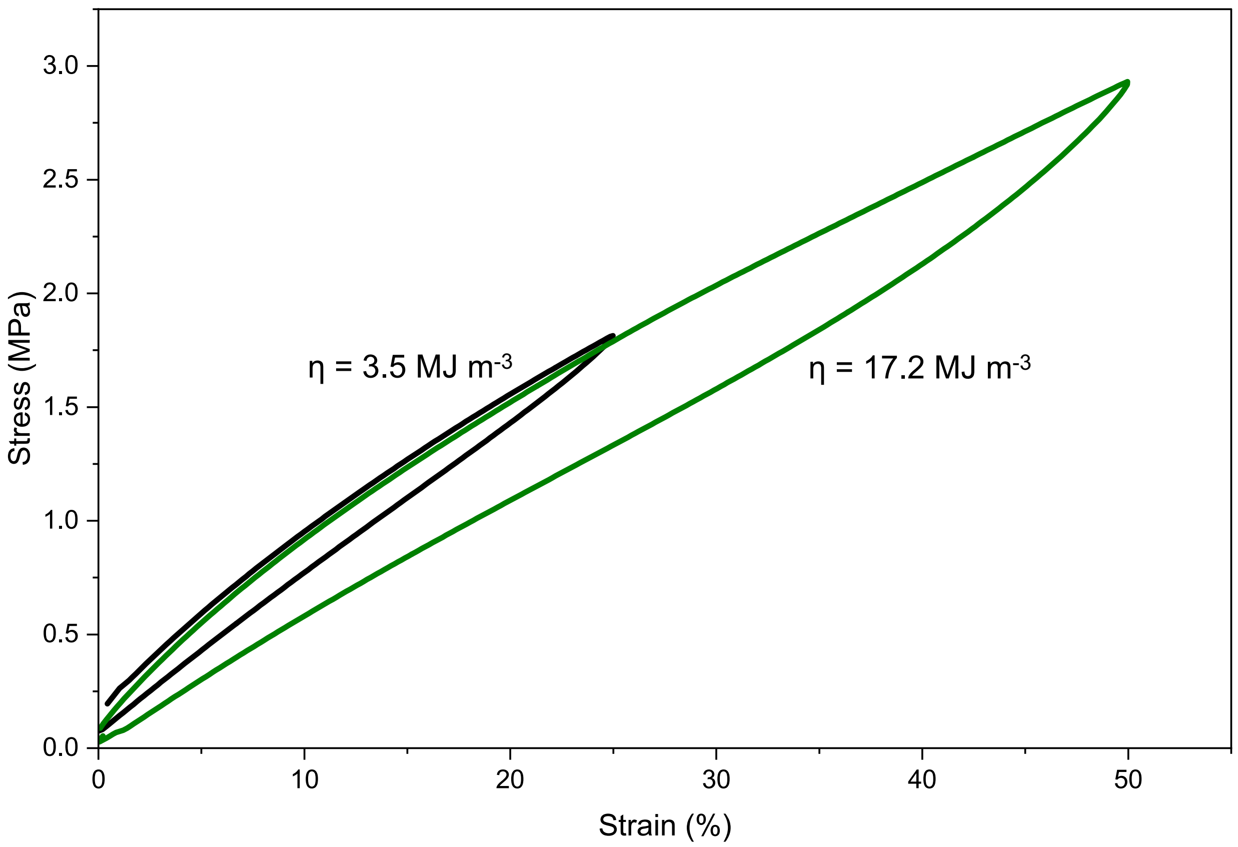

Figure S42. Continuous loading-unloading tensile curves of $P3HB_{[18/42/40]}$ at 25% strain (black) and 50% strain (green); η = energy loss coefficient.

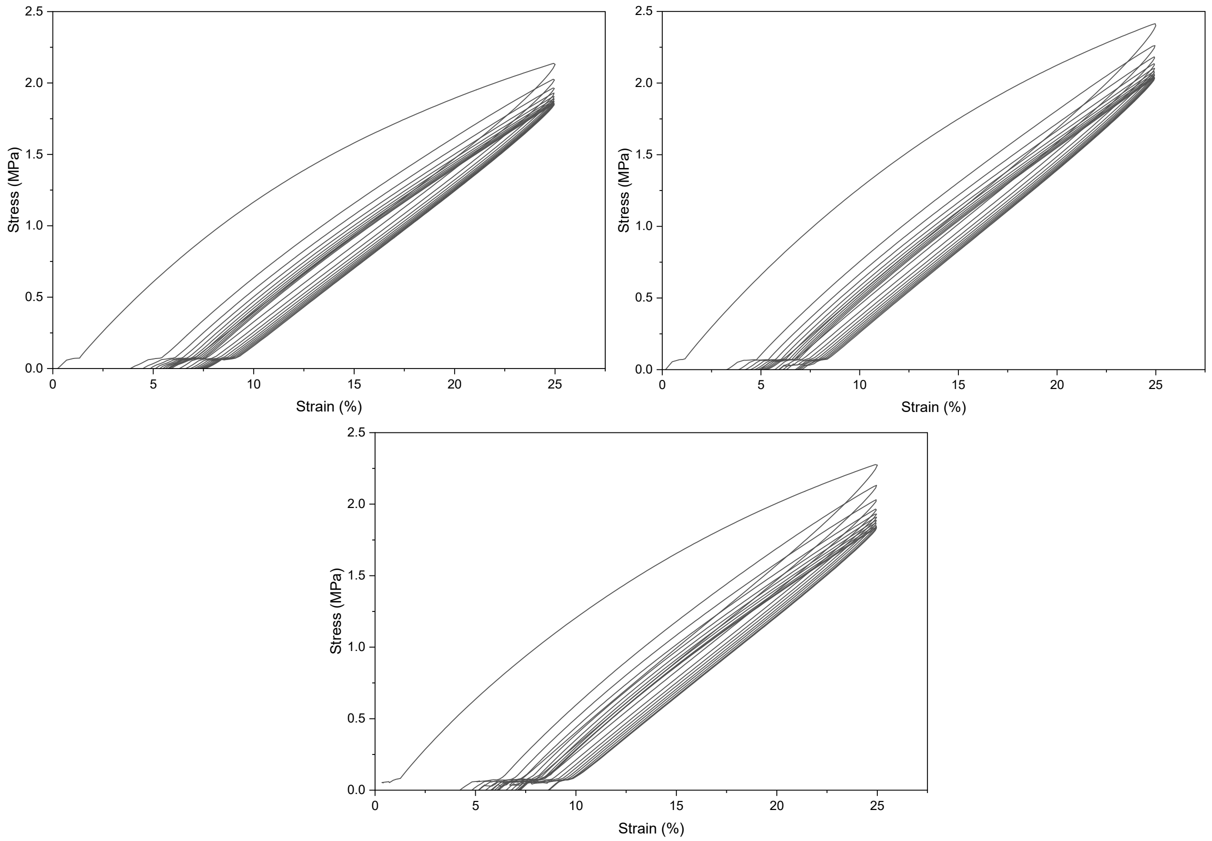

Figure S43. Hysteresis of $P3HB_{[18/42/40]}$ with 10 loading-unloading cycles at a strain of 25% without any resting time; run in triplicate on three distinct samples.

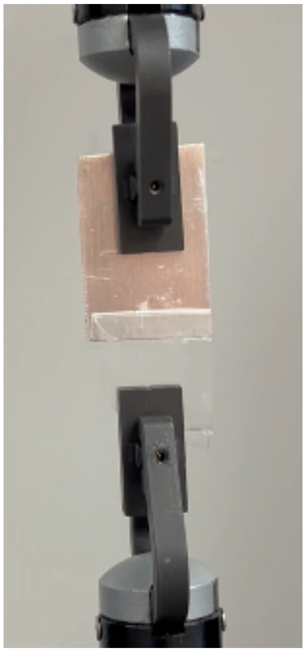

Figure S44. Photo of Tape 1 mounted on an Instron for peel testing.

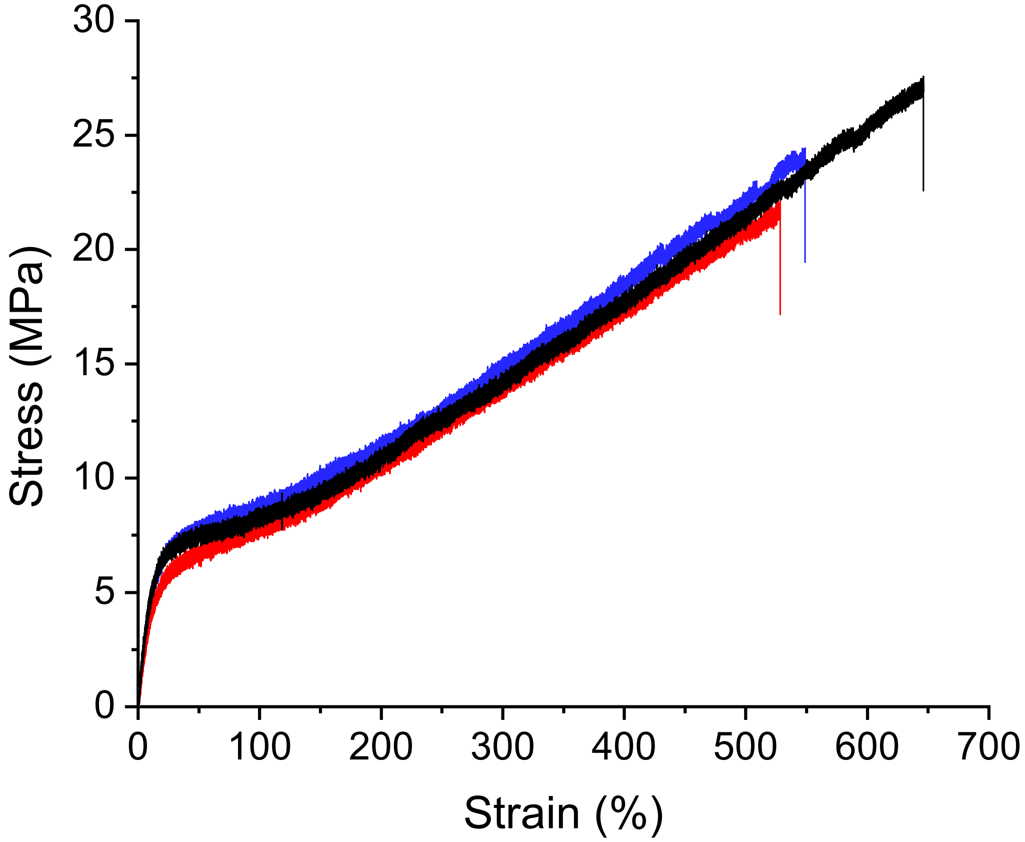

Figure S45. Triplicate stress-strain curves of recycled Tape 1.

Supplementary Tables

Table S1. Shift factors analyzed from the master curve.

P3HB polymer	WLF equation			Arrhenius equation	
	c_1	c_2 (K)	R^2	E_A (kJ/mol)	R^2
$P3HB_{[29/40/31]}$	7.30	81.33	0.99	92.97	0.95
$P3HB_{[33/41/26]}$	7.21	80.27	0.99	91.98	0.95
$P3HB_{[41/39/20]}$	7.74	90.55	0.99	93.67	0.96

Reference temperature T_0 = 303K

Table S2. Plateau modulus and the entanglement molecular weight analyzed from master curve.

P3HB	G_N^0 (MPa)	M_e (g/mol)
$P3HB_{[41/39/20]}$	0.36	7417
$P3HB_{[33/41/26]}$	0.41	6513
$P3HB_{[29/40/31]}$	0.43	6210

Table S3. Carreau model parameters fitted by the viscosity measurement.

P3HB polymer	η_0 (Pa s)	η_∞ (Pa s)	k (s)	n	R^2
$P3HB_{[29/40/31]}$	253	1.40×10^{-3}	1.45×10^{-2}	-4.18	0.97
$P3HB_{[33/41/26]}$	416	4.60×10^{-3}	1.27×10^{-2}	-5.41	0.99
$P3HB_{[41/39/20]}$	433	1.40×10^{-3}	1.18×10^{-2}	-6.59	0.99

Table S4. Triplicate tensile data for $P3HB_{[0/41/59]}$.

Sample	Young's Modulus (E, MPa)	Standard Dev. (±)	Tensile strength (σ, MPa)	Standard Dev. (±)	Elongation at Break (ε, %)	Standard Dev. (±)	Toughness (U_t, MJ m^{-3})	Standard Dev. (±)
1	339		35.0		347		84.2	
2	271		29.1		355		71.8	
3	307		30.2		361		75.0	
Average	306	34	31.4	3.1	354	6.5	77.0	6.4

Table S5. Triplicate tensile data for $P3HB_{[4/39/57]}$.

Sample	Young's Modulus (E, MPa)	Standard Dev. (±)	Tensile strength (σ, MPa)	Standard Dev. (±)	Elongation at Break (ε, %)	Standard Dev. (±)	Toughness (U_t, MJ m^{-3})	Standard Dev. (±)
1	87.4		31.6		470		94.3	
2	97.0		31.5		481		97.2	
3	111		30.0		477		89.9	
Average	98.6	12	31.0	0.9	476	5.3	93.8	3.7

Table S6. Triplicate tensile data for $P3HB_{[10/45/45]}$.

Sample	Young's Modulus (E, MPa)	Standard Dev. (±)	Tensile strength (σ, MPa)	Standard Dev. (±)	Elongation at Break (ε, %)	Standard Dev. (±)	Toughness (U_t, MJ m^{-3})	Standard Dev. (±)
1	20.4		13.9		450		36.3	
2	14.1		17.7		588		59.8	
3	9.22		17.5		552		52.7	
Average	14.6	5.6	16.4	2.1	530	72	49.6	12

Table S7. Triplicate tensile data for $P3HB_{[18/42/40]}$.

Sample	Young's Modulus (*E*, MPa)	Standard Dev. (±)	Tensile strength (σ, MPa)	Standard Dev. (±)	Elongation at Break (ε, %)	Standard Dev. (±)	Toughness (U_T, MJ m^{-3})	Standard Dev. (±)
1	14.8		15.0		676		53.0	
2	15.2		15.2		673		57.9	
3	15.6		15.1		676		55.9	
Average	15.2	0.41	15.1	0.1	675	1.5	55.6	2.4

Table S8. Triplicate tensile data for $P3HB_{[49/37/14]}$.

Sample	Young's Modulus (*E*, MPa)	Standard Dev. (±)	Tensile strength (σ, MPa)	Standard Dev. (±)	Elongation at Break (ε, %)	Standard Dev. (±)	Toughness (U_T, kJ m^{-3})	Standard Dev. (±)
1	0.6		4.2		525		9.1	
2	1.7		4.0		360		7.8	
3	1.8		4.1		356		8.1	
Average	1.4	0.6	4.1	0.1	414	96	8.3	0.7

Table S9. Triplicate tensile data for $P3HB_{[57/34/9]}$.

Sample	Young's Modulus (*E*, MPa)	Standard Dev. (±)	Tensile strength (σ, MPa)	Standard Dev. (±)	Elongation at Break (ε, %)	Standard Dev. (±)	Toughness (U_T, MJ m^{-3})	Standard Dev. (±)
1	27.5		13.5		859		33.9	
2	21.7		10.5		908		38.4	
3	29.7		8.1		554		28.6	
Average	26.3	4.1	10.7	2.7	774	192	33.4	4.9

Table S10. Triplicate tensile data for $P3HB_{[63/32/5]}$.

Sample	Young's Modulus (*E*, MPa)	Standard Dev. (±)	Tensile strength (σ, MPa)	Standard Dev. (±)	Elongation at Break (ε, %)	Standard Dev. (±)	Toughness (U_T, MJ m^{-3})	Standard Dev. (±)
1	288		8.6		530		31.0	
2	291		8.5		525		31.1	
3	304		11.5		602		44.3	
Average	294	8.4	9.5	1.7	552	43	35.5	7.6

Table S11. Triplicate tensile data for recycled Tape 1.

Sample	Young's Modulus (*E*, MPa)	Standard Dev. (±)	Tensile strength (σ, MPa)	Standard Dev. (±)	Elongation at Break (ε, %)	Standard Dev. (±)	Toughness (U_T, MJ m^{-3})	Standard Dev. (±)
1	47.5		22.1		528		68.1	
2	45.0		27.6		646		99.8	
3	37.8		24.4		549		78.3	
Average	43.4	5.0	24.7	2.3	574	51.4	82.1	13.2

Table S12. Adhesion data of adhesive P3HBs.

Sample	Run 1 (MPa)	Run 2 (MPa)	Run 3 (MPa)	Average (MPa)	Standard Deviation
$P3HB_{[29/40/31]}$	1.27	1.06	1.22	1.2	0.1
$P3HB_{[33/41/26]}$	0.63	0.57	0.58	0.59	0.03
$P3HB_{[41/39/20]}$	0.67	0.54	0.57	0.59	0.06
$P3HB_{[49/37/14]}$	1.80	2.27	2.08	2.1	0.2

Table S13. Peel testing data of manufactured tapes.

Sample	Run 1 (MPa)	Run 2 (MPa)	Run 3 (MPa)	Average (MPa)	Standard Deviation
Tape 1	0.38	0.36	0.33	0.36	0.03
Tape 2	0.22	0.15	0.18	0.19	0.04

Table S14. Summary of estimated lifetime of P3HB samples in freshwater environments using first order kinetic model.

Sample	Environment	Rate constant (day^{-1})	Estimated 90% biodegradation time (day)	R^2
$P3HB_{[80/20/0]}$	Freshwater	0.006153	273	0.6643
$P3HB_{[10/45/45]}$	Freshwater	0.013505	131	0.7508
$P3HB_{[33/41/26]}$	Freshwater	0.004234	529	0.9206

Table S15. Result of *meso*-$8DL^{Me}$ polymerization[a].

[*meso*-$8DL^{Me}$]/[1]/[BnOH]	Time (min.)	Conversion (%)
100/1/1	0.5	100

[a] Polymerization conditions: 0.581 mmol *meso*-$8DL^{Me}$ was dissolved in 1.5 M dichloromethane at 25 °C. 0.00581 mmol **1** and 0.00581 mmol BnOH were allowed to undergo *in situ* alcoholysis and added to the monomer solution. Conversion was monitored using ^1H NMR of the quenched solution in benzoic acid/chloroform (5% w/v benzoic acid).

Table S16. Result of *rac*-$8DL^{Me}$ polymerization[a].

[*rac*-$8DL^{Me}$]/[1]/[BnOH]	Time (min.)	Conversion (%)
100/1/1	0.5	53
〃	1	71
〃	1.5	84
〃	2	91
〃	5	100

[a] Polymerization conditions: 0.581 mmol *rac*-$8DL^{Me}$ was dissolved in 1.5 M dichloromethane at 25 °C. 0.00581 mmol **1** and 0.00581 mmol BnOH were allowed to undergo *in situ* alcoholysis and added to the monomer solution. Conversion was monitored using ^1H NMR of the quenched solution in benzoic acid/chloroform (5% w/v benzoic acid).

Table S17. Result of *meso*-8DL^{Me}/*rac*-8DL^{Me} copolymerizations[a].

***meso*:*rac* (8DL^{Me})**	**P3HB**	**Time (min.)**	**Conversion**
100:0	$P3HB_{[0/41/59]}$	2	>99
90:10	$P3HB_{[4/39/57]}$	105	>99
80:20	$P3HB_{[10/45/45]}$	260	94
70:30	$P3HB_{[18/42/40]}$	60	94
60:40	$P3HB_{[29/40/31]}$[b]	300	98
50:50	$P3HB_{[33/41/26]}$	345	96
40:60	$P3HB_{[41/39/20]}$	655	96
30:70	$P3HB_{[49/37/14]}$	960	>99
20:80	$P3HB_{[57/34/9]}$	210	94
10:90	$P3HB_{[63/32/5]}$	180	95
0:100	$P3HB_{[80/20/0]}$[c]	600	>99

[a] Polymerization conditions and conversion monitoring described in the Materials and Methods section. [b] Data was previously reported.[45] [c] Data was previously reported.[42]

Supplementary Note

The crystallinity of the resulting P3HB was calculated using the equation X_c (%) = $(\Delta H_f/\Delta H_f^0) \cdot 100$, where ΔH_f and ΔH_f^0 is the heat of fusion (J g^{-1}) of the synthesized P3HB and the 100% crystalline P3HB (146 J g^{-1})[60] respectively.

Bibliography

[39] X. Tang, E. Y.-X. Chen, "Chemical Synthesis of Perfectly Isotactic and High Melting Bacterial Poly(3-Hydroxybutyrate) from Bio-Sourced Racemic Cyclic Diolide." *Nat. Commun.,* **2018**, *9*, 2345.

[42] Z. Zhang, E. C. Quinn, J. L. Olmedo-Martínez, M. R. Caputo, K. A. Franklin, A. J. Müller, E. Y.-X. Chen, "Toughening Brittle Bio-P3HB with Synthetic P3HB of Engineered Stereomicrostructures." *Angew. Chem. Int. Ed.,* **2023**, *62*, e202311264.

[48] X. Tang, A. H. Westlie, E. M. Watson, E. Y.-X. Chen, "Stereosequenced Crystalline Polyhydroxyalkanoates from Diastereomeric Monomer Mixtures." *Science*, **2019**, *366*, 754-758.

[55] A. Eckstein, J. Suhm, C. Friedrich, R. D. Maier, J. Sassmannshausen, M. Bochmann, R. Mülhaupt, "Determination of Plateau Moduli and Entanglement Molecular Weights of Isotactic, Syndiotactic, and Atactic Polypropylenes Synthesized with Metallocene Catalysts." *Macromolecules*, **1998**, *31*, 1335-1340.

[56] S. Ebnesajjad, "Plastic Films in Food Packaging: Materials, Technology and Applications." *PDL Handbook Series*, William Andrew Publishing, Oxford, **2013.**

[57] Z. Zhu, P. Dakwa, P. Tapadia, R. S. Whitehouse, S.-Q. Wang, "Rheological Characterization of Flow and Crystallization Behavior of Microbial Synthesized Poly(3-hydroxybutyrate-co-4-hydroxybutyrate)." *Macromolecules*, **2003**, *36*, 4891-4897.

[58] S. H. Park, S. T. Lim, T. K. Shin, H. J. Choi, M. S. Jhon, "Viscoelasticity of Biodegradable Polymer Blends of Poly(3-Hydroxybutyrate) and Poly(Ethylene Oxide)." *Polymer*, **2001**, *42*, 5737-5742.

[59] ISO 14851:2019, Determination of the Ultimate Aerobic Biodegradability of Plastic Materials in an Aqueous Medium—Method by Measuring the Oxygen Demand in a Closed Respirometer, International Organization for Standardization, Geneva, **2019**.

[60] P. J. Barham, A. Keller, E. L. Otun, P. A. Holmes, "Crystallization and Morphology of a Bacterial Thermoplastic: Poly-3-Hydroxybutyrate." *J. Mater. Sci.,* **1984**, *19*, 2781-2794.
